# Supplementary material for: Free Radical Isomerizations in Acetylene Bromoboration Reaction
Source: Molecules. 2021 Apr 25;26(9):2501. doi: 10.3390/molecules26092501 (PMC8123272; doi:10.3390/molecules26092501)
Supplement: Supplementary file 1 [file molecules-26-02501-s001.zip › molecules-1170615-supplementary.pdf]

Supporting Information for:

Free Radical Isomerizations in Acetylene Bromoboration Reaction

Hugo Semrád [1], Ctibor Mazal [1], and Markéta Munzarová [1,*]

Department of Chemistry, Faculty of Science, Masaryk University, Kotlářská 2, 611 37 Brno, Czech Republic
* Correspondence: marketa@chemi.muni.cz

Table of Contents

1. **Reaction profiles for polar addition-elimination isomerization: via interaction of HBr with the C=C bond S2**
2. **Reaction profiles for HBr interaction with B–C bond S3**
3. **Influence of ZPE determined with diffuse basis sets on Gibbs energy profile S4**
4. **Transition state and intermediate structures related to Figures 9, 10 and S7........ S4**
5. **Absolute energy contributions and imaginary frequencies S5**
6. **Optimized cartesian coordinates S10**
   - 6.1 B3LYP-GD3BJ cartesian coordinates referring to Figure 1. (a) S10
   - 6.2 MP2 cartesian coordinates referring to Figure 1. (b)........S12
   - 6.3 B3LYP-GD3BJ cartesian coordinates referring to Figures 2 and 3 ....S14
   - 6.4 MP2 cartesian coordinates referring to Figures 4 and 5 ....S21
   - 6.5 MP2 cartesian coordinates referring to Figures 6 and 7 ....S26
   - 6.6 B3LYP-GD3BJ cartesian coordinates referring to Figure 8........S31
   - 6.7 MP2 cartesian coordinates referring to Figures 9 and 10 ....S34
   - 6.8 B3LYP-GD3BJ cartesian coordinates referring to Figure 11........S41

7. Vibrational frequencies for (*E*)-1 + Br$^\bullet$ and ^{add}(*E*)-1 + Br$^\bullet$........S45

1. Reaction profiles for polar addition-elimination isomerization: via interaction of HBr with the C=C bond

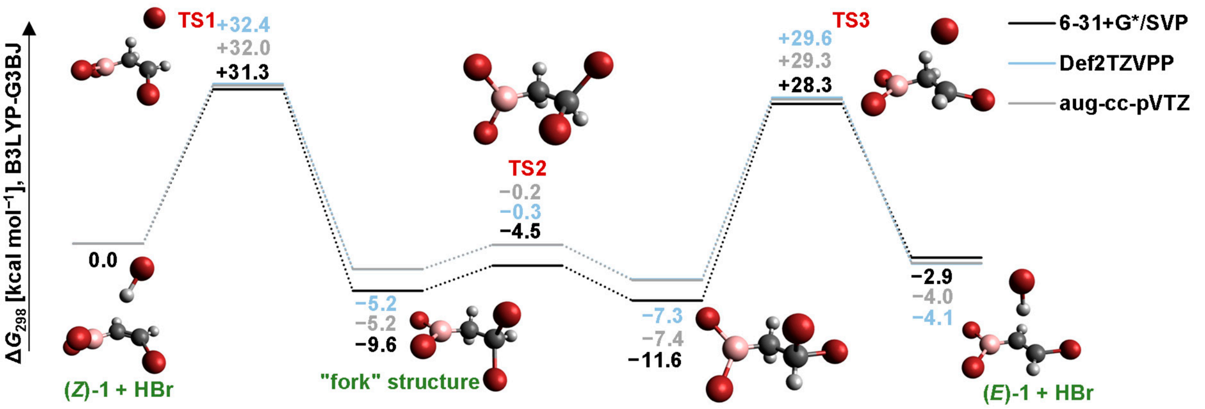

Figure S1. Dispersion corrected B3LYP results for polar HBr addition to (*Z*)-1 double bond followed by the rotation and (*E*)-1 formation.

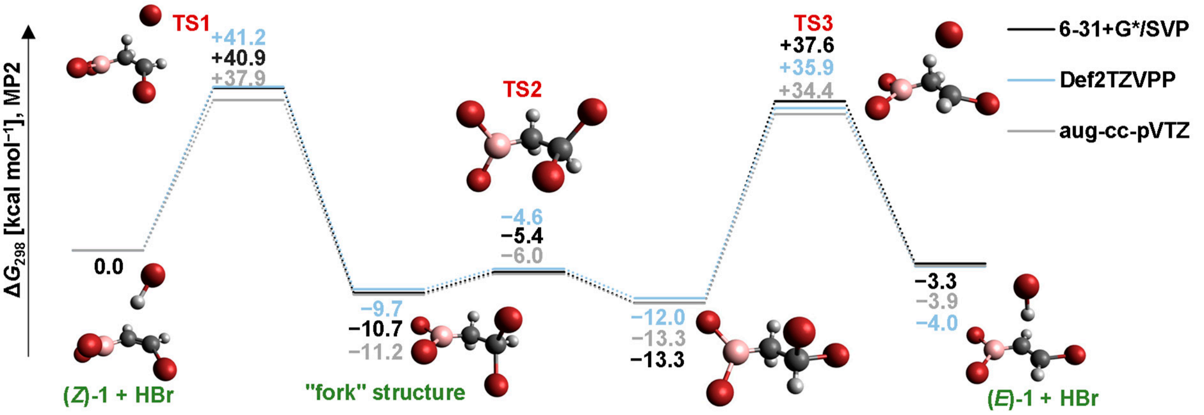

Figure S2. MP2 results for polar HBr addition to (*Z*)-1 double bond followed by the rotation and (*E*)-1 formation.

2. Reaction profiles for HBr interaction with B–C bond

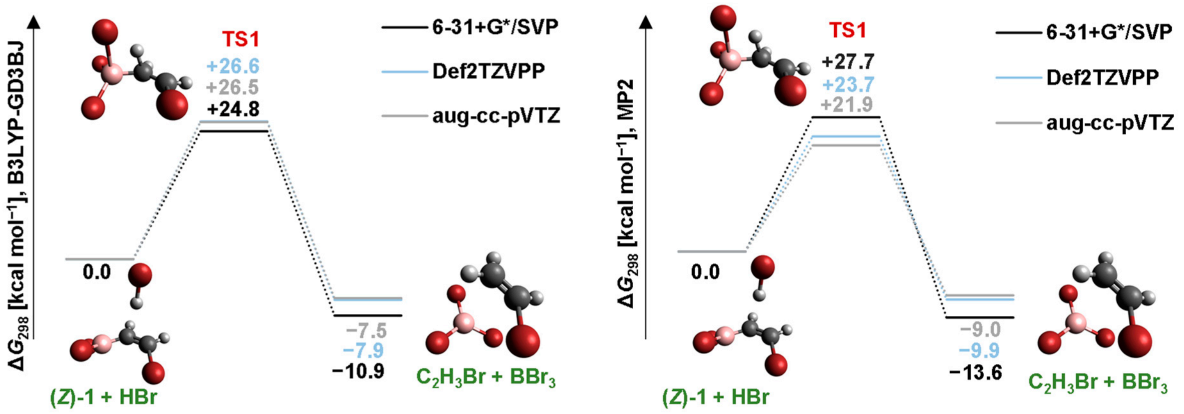

Figure S3. Dispersion corrected B3LYP (left) and MP2 (right) possible reaction pathway given as an HBr addition to (*Z*)-1 and its decomposition to vinylbromide and boron tribromide.

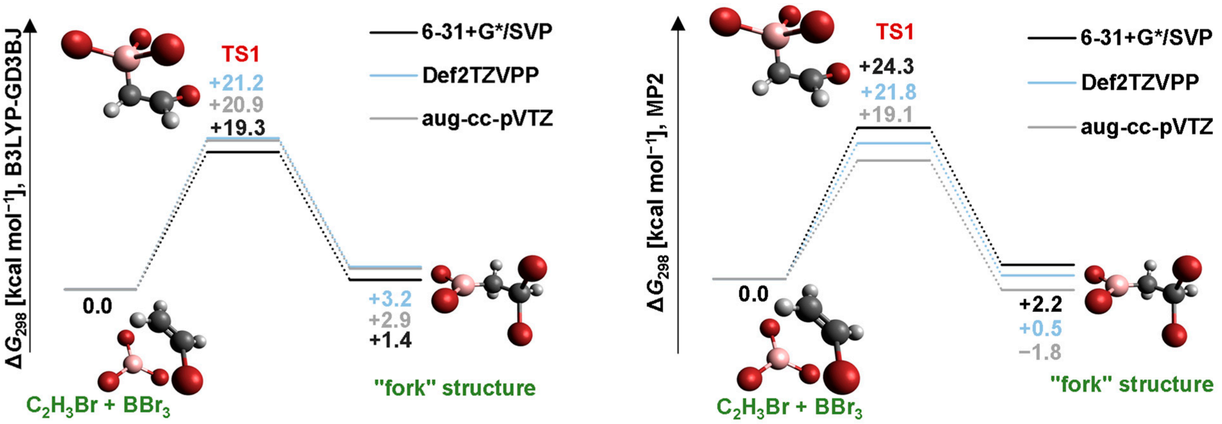

Figure S4. Dispersion corrected B3LYP (left) and MP2 (right) possible reaction pathway given as a vinylbromide and boron tribromide reaction to form a “fork structure”.

3. Influence of ZPE determined with diffuse basis sets on Gibbs energy profile

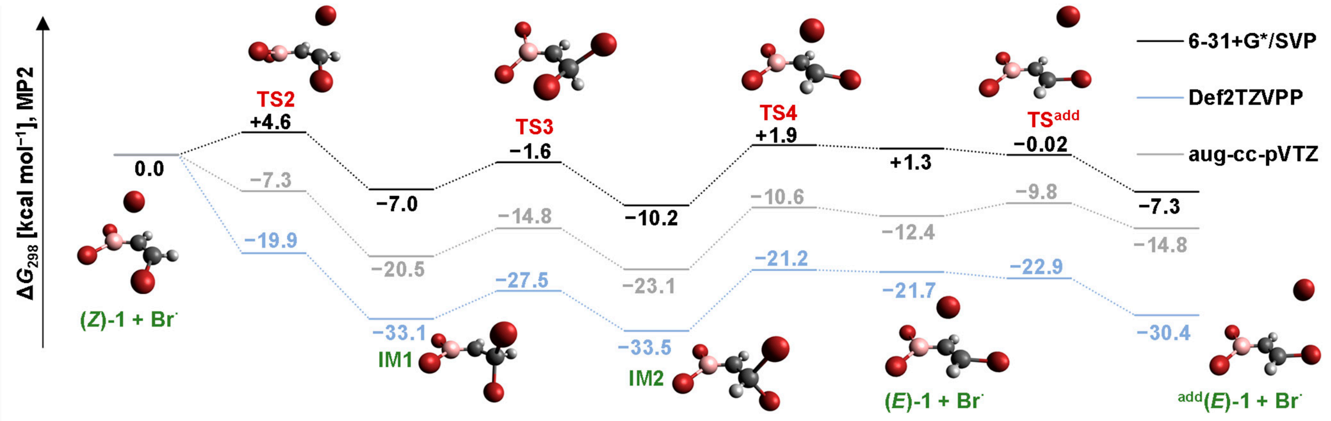

Figure S5. MP2 Gibbs Free Energy profile related to Figure 5 with ZPE, thermal and entropy contributions calculated with same basis sets as the respective electron energy contributions.

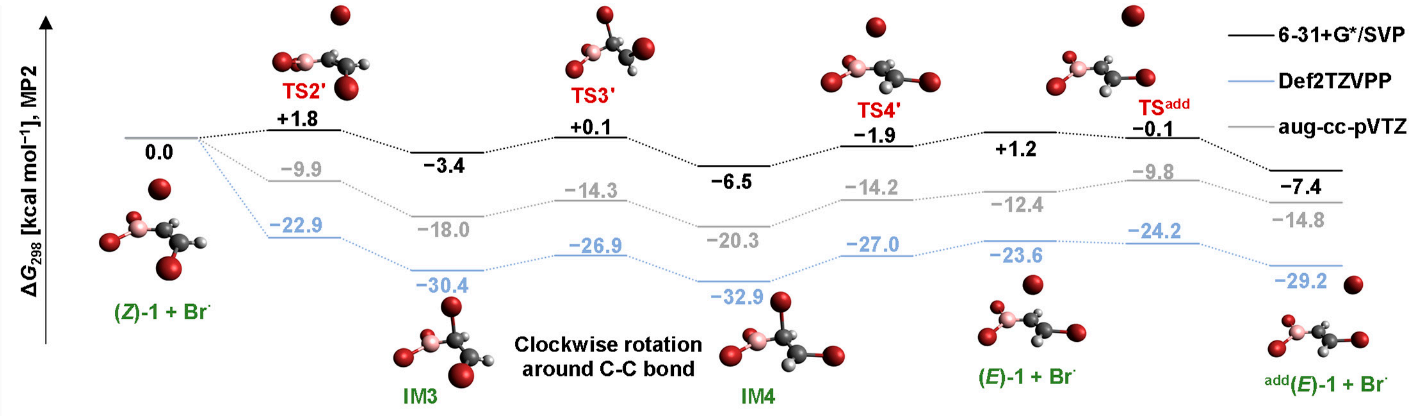

Figure S6. MP2 Gibbs Free Energy profile related to Figure 7 with ZPE, thermal and entropy contributions calculated with same basis sets as the respective electron energy contributions.

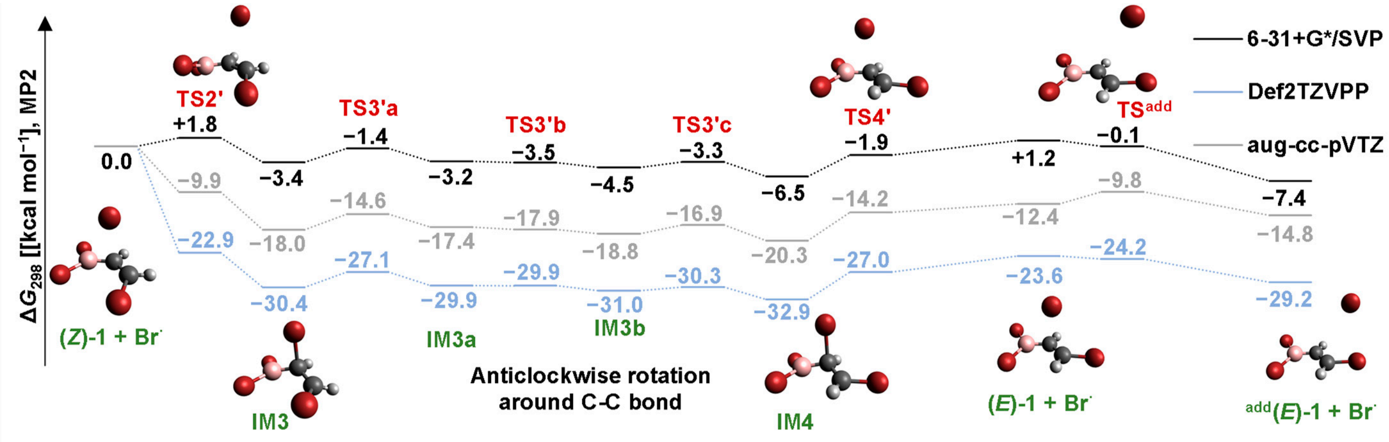

Figure S7. MP2 Gibbs Free Energy profile related to Figure 10 with ZPE, thermal and entropy contributions calculated with same basis sets as the respective electron energy contributions.

4. Transition state and intermediate structures related to Figures 9, 10 and S7.

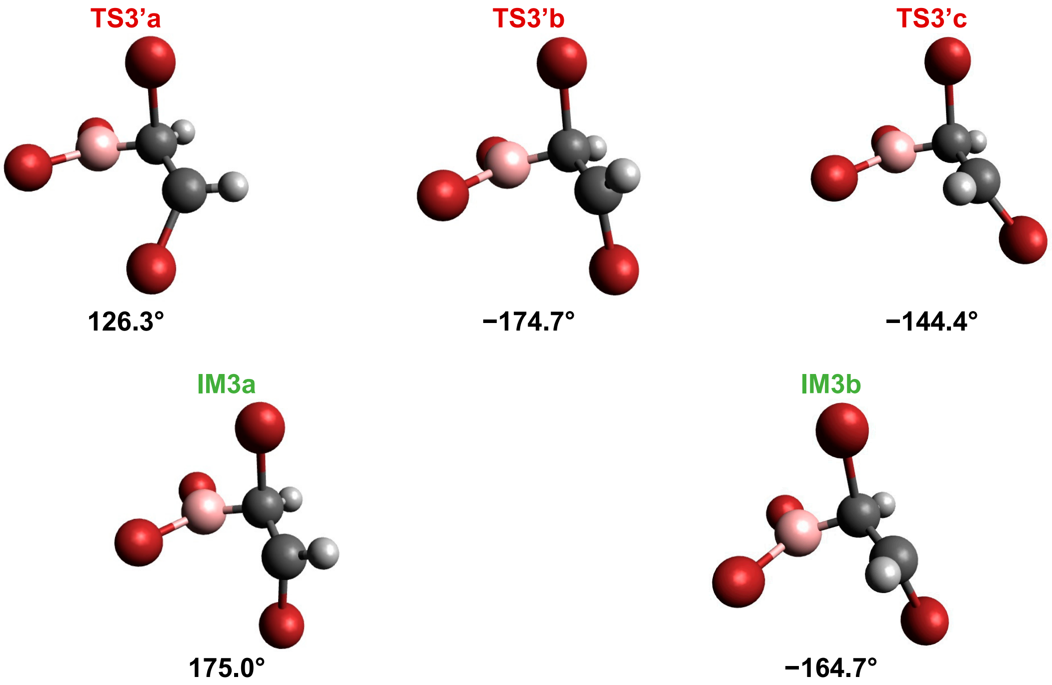

Figure S8. Structures with torsion angle values defined by Br–C(B)–C(Br)–Br related to Figure 9, 10 and S7.

5. Absolute energy contributions and imaginary frequencies

Table S1: Electronic energies, zero-point corrections, thermal corrections to enthalpy and to Gibbs Free energy at 298 K (in Hartrees per particle) and imaginary frequencies (in cm^{-1}).

**Figure 1. (a) B3LYP-GD3BJ**	E_{el}	**ZPE corrections**	H_{corr}	G_{corr}	**Imaginary frequency**
**C_2H_2 + BBr_3**					
6-31+G* (B, C, H)/SVP (Br)	−7823.93146443	0.033717	0.044108	−0.005987	
Def2TZVPP	−7824.90656498	0.033854	0.044192	−0.005787	–
aug-cc-pVTZ	−7825.04458519	0.033769	0.044127	−0.005940	
6-31+G* (B, C, H)/ECP28MWB (Br)	−142.441487350	0.033549	0.043957	−0.006268	
**TS1**					
6-31+G* (B, C, H)/SVP (Br)	−7823.91636534	0.033966	0.042581	−0.001225	−81.2
Def2TZVPP	−7824.88741625	0.033938	0.042497	−0.001164	−122.2
aug-cc-pVTZ	−7825.02593516	0.033893	0.042453	−0.001209	−123.9
6-31+G* (B, C, H)/ECP28MWB (Br)	−142.429489978	0.033661	0.042816	−0.002348	−207.8
**(Z)-1**					
6-31+G* (B, C, H)/SVP (Br)	−7823.96989499	0.038729	0.047010	0.002530	
Def2TZVPP	−7824.94015522	0.038575	0.046850	0.002476	–
aug-cc-pVTZ	−7825.07819873	0.038535	0.046818	0.002425	
6-31+G* (B, C, H)/ECP28MWB (Br)	−142.479194496	0.038507	0.046823	0.002407	
**Figure 1. (b) MP2**	E_{el}	**ZPE corrections**	H_{corr}	G_{corr}	**Imaginary frequency**
**C_2H_2 + BBr_3**					
6-31+G* (B, C, H)/SVP (Br)	−7818.95558150	0.032807	0.043416	−0.007083	
Def2TZVPP	−7820.14420512	0.033431	0.043866	−0.006603	–
aug-cc-pVTZ	−7820.16710939	0.033640	0.043853	−0.004978	
**TS1**					
6-31+G* (B, C, H)/SVP (Br)	−7818.92724553	0.034899	0.043208	0.000114	−333.4
Def2TZVPP	−7820.11621963	0.034788	0.043092	0.000023	−364.9
aug-cc-pVTZ	−7820.14206298	0.034532	0.042870	−0.000257	−363.4
**(Z)-1**					
6-31+G* (B, C, H)/SVP (Br)	−7818.98233686	0.038945	0.047251	0.001634	
Def2TZVPP	−7820.17144089	0.039001	0.047233	0.002770	–
aug-cc-pVTZ	−7820.19513974	0.038893	0.047150	0.002534	
**Figure 2&3 B3LYP-GD3BJ**	E_{el}	**ZPE corrections**	H_{corr}	G_{corr}	**Imaginary frequency**
**(Z)-1 + Br$^{\bullet}$**					
6-31+G* (B, C, H)/SVP (Br)	−10397.8224825	0.038390	0.049180	−0.003452	
Def2TZVPP	−10399.1041085	0.038244	0.049000	−0.003341	–
aug-cc-pVTZ	−10399.2890225	0.038219	0.048999	−0.003640	
6-31+G* (B, C, H)/ECP28MWB (Br)	−155.841389135	0.038732	0.049438	−0.003067	
**TS2**					
6-31+G* (B, C, H)/SVP (Br)	−10397.8163961	0.038340	0.048285	−0.003000	−219.0
Def2TZVPP	−10399.0979040	0.038120	0.048055	−0.002985	−221.4
aug-cc-pVTZ	−10399.2829100	0.038072	0.048018	−0.003120	−219.3
6-31+G* (B, C, H)/ECP28MWB (Br)	−155.835830707	0.038041	0.048005	−0.002774	−228.1
**IM1**					
6-31+G* (B, C, H)/SVP (Br)	−10397.8240432	0.038908	0.049306	−0.002460	
Def2TZVPP	−10399.1046616	0.038646	0.049079	−0.002898	–
aug-cc-pVTZ	−10399.2894493	0.038606	0.049048	−0.002943	
6-31+G* (B, C, H)/ECP28MWB (Br)	−155.839475847	0.038650	0.049137	−0.002781	
**TS3**					
6-31+G* (B, C, H)/SVP (Br)	−10397.8163346	0.038521	0.047921	−0.000793	−33.2
Def2TZVPP	−10399.0970264	0.038210	0.047625	−0.001075	−31.2
aug-cc-pVTZ	−10399.2818926	0.038177	0.047595	−0.001070	−31.0
6-31+G* (B, C, H)/ECP28MWB (Br)	−155.831278826	0.038113	0.047647	−0.001729	−44.5
**IM2**					
6-31+G* (B, C, H)/SVP (Br)	−10397.8284975	0.039482	0.049698	−0.002281	
Def2TZVPP	−10399.1088665	0.039153	0.049413	−0.003499	–
aug-cc-pVTZ	−10399.2937301	0.039118	0.049383	−0.003299	
6-31+G* (B, C, H)/ECP28MWB (Br)	−155.843369520	0.039278	0.049569	−0.002349	
**TS4**					

6-31+G* (B, C, H)/SVP (Br)	−10397.8204562	0.038342	0.048330	−0.002570	−231.3
Def2TZVPP	−10399.1018716	0.038109	0.048108	−0.002813	−233.7
aug-cc-pVTZ	−10399.2868418	0.038067	0.048070	−0.002844	−232.1
6-31+G* (B, C, H)/ECP28MWB (Br)	−155.839561730	0.038144	0.048155	−0.002740	−230.5
**(*E*)-1 + Br$^{\bullet}$**					
6-31+G* (B, C, H)/SVP (Br)	−10397.8270080	0.038575	0.049342	−0.002938	
Def2TZVPP	−10399.1087867	0.038513	0.049244	−0.002868	–
aug-cc-pVTZ	−10399.2937139	0.038397	0.049149	−0.003061	
6-31+G* (B, C, H)/ECP28MWB (Br)	−155.846221404	0.038723	0.049501	−0.002956	
**TS^{add}**					
6-31+G* (B, C, H)/SVP (Br)	−10397.8124274	0.038440	0.048672	−0.003568	−245.5
Def2TZVPP	−10399.0954649	0.038161	0.048447	−0.004224	−296.3
aug-cc-pVTZ	−10399.2804659	0.038255	0.048478	−0.003798	−267.3
6-31+G* (B, C, H)/ECP28MWB (Br)	−155.835056095	0.038086	0.048475	−0.004877	−235.0
**^{add}(*E*)-1 + Br$^{\bullet}$**					
6-31+G* (B, C, H)/SVP (Br)	−10397.8179419	0.039031	0.049940	−0.004331	
Def2TZVPP	−10399.1023815	0.038747	0.049738	−0.005651	–
aug-cc-pVTZ	−10399.2875955	0.038875	0.049736	−0.004316	
6-31+G* (B, C, H)/ECP28MWB (Br)	−155.839701763	0.038776	0.049763	−0.005399	
**Figure 4&5 MP2**	E_{el}	**ZPE corrections**	H_{corr}	G_{corr}	**Imaginary frequency**
**(*Z*)-1 + Br$^{\bullet}$**					
6-31+G* (B, C, H)/SVP (Br)	−10391.2829261	0.046368	0.056636	0.004194	
Def2TZVPP	−10392.8292079	0.084352	0.094441	0.042928	–
aug-cc-pVTZ	−10392.8609393	0.063139	0.073188	0.022003	
**TS2**					
6-31+G* (B, C, H)/SVP (Br)	−10391.2693103	0.039399	0.049231	−0.002160	−422.5
Def2TZVPP	−10392.8169888	0.039421	0.049157	−0.000948	−463.7
aug-cc-pVTZ	−10392.8493827	0.039263	0.049029	−0.001232	−476.5
**IM1**					
6-31+G* (B, C, H)/SVP (Br)	−10391.2889014	0.039935	0.050157	−0.001014	
Def2TZVPP	−10392.8380002	0.039731	0.049911	−0.000984	–
aug-cc-pVTZ	−10392.8706482	0.039661	0.049845	−0.000956	
**TS3**					
6-31+G* (B, C, H)/SVP (Br)	−10391.2819150	0.039760	0.048960	0.000719	−30.9
Def2TZVPP	−10392.8305801	0.039447	0.048620	0.000549	−19.3
aug-cc-pVTZ	−10392.8631077	0.039318	0.048503	0.000551	−26.9
**IM2**					
6-31+G* (B, C, H)/SVP (Br)	−10391.2937849	0.040454	0.050510	−0.001203	
Def2TZVPP	−10392.8421277	0.040204	0.050218	−0.000762	–
aug-cc-pVTZ	−10392.8745680	0.040104	0.050148	−0.001204	
**TS4**					
6-31+G* (B, C, H)/SVP (Br)	−10391.2746248	0.039441	0.049287	−0.001010	−444.4
Def2TZVPP	−10392.8225667	0.039345	0.049129	−0.000744	−476.0
aug-cc-pVTZ	−10392.8549795	0.039218	0.049009	−0.000874	−483.0
**(*E*)-1 + Br$^{\bullet}$**					
6-31+G* (B, C, H)/SVP (Br)	−10391.2890588	0.053829	0.064107	0.012340	
Def2TZVPP	−10392.8349987	0.052312	0.062500	0.011043	–
aug-cc-pVTZ	−10392.8667630	0.049099	0.059247	0.008116	
**TS^{add}**					
6-31+G* (B, C, H)/SVP (Br)	−10391.2837031	0.047129	0.057201	0.004939	−14.5
Def2TZVPP	−10392.8272627	0.044610	0.054674	0.002367	−23.3
aug-cc-pVTZ	−10392.8565891	0.043752	0.053768	0.002085	−26.6
**^{add}(*E*)-1 + Br$^{\bullet}$**					
6-31+G* (B, C, H)/SVP (Br)	−10391.2849680	0.039204	0.050302	−0.005458	
Def2TZVPP	−10392.8285995	0.039304	0.050230	−0.004262	–
aug-cc-pVTZ	−10392.8583305	0.039330	0.050247	−0.004237	
**Figure 6&7 MP2**	E_{el}	**ZPE corrections**	H_{corr}	G_{corr}	**Imaginary frequency**

**(*Z*)-1 + Br$^{\bullet}$**					
6-31+G* (B, C, H)/SVP (Br)	−10391.2829261	0.046368	0.056636	0.004194	
Def2TZVPP	−10392.8292079	0.084352	0.094441	0.042928	–
aug-cc-pVTZ	−10392.8609393	0.063139	0.073188	0.022003	
**TS2′**					
6-31+G* (B, C, H)/SVP (Br)	−10391.2748451	0.039907	0.049862	−0.001010	−163.7
Def2TZVPP	−10392.8223098	0.040036	0.049902	−0.000393	−195.5
aug-cc-pVTZ	−10392.8543104	0.039970	0.049840	−0.000371	−230.7
**IM3**					
6-31+G* (B, C, H)/SVP (Br)	−10391.2828917	0.039452	0.049745	−0.001270	
Def2TZVPP	−10392.8328423	0.038920	0.049276	−0.001820	–
aug-cc-pVTZ	−10392.8654458	0.038803	0.049205	−0.002109	
**TS3′**					
6-31+G* (B, C, H)/SVP (Br)	−10391.2777843	0.038704	0.048323	−0.000733	−62.5
Def2TZVPP	−10392.8281537	0.038336	0.047966	−0.000996	−57.5
aug-cc-pVTZ	−10392.8606874	0.038261	0.047913	−0.001099	−60.2
**IM4**					
6-31+G* (B, C, H)/SVP (Br)	−10391.2877791	0.039608	0.049892	−0.001269	
Def2TZVPP	−10392.8374572	0.039269	0.049534	−0.001283	–
aug-cc-pVTZ	−10392.8699920	0.039210	0.049477	−0.001316	
**TS4′**					
6-31+G* (B, C, H)/SVP (Br)	−10391.2811989	0.039903	0.049853	−0.000529	−183.4
Def2TZVPP	−10392.8285547	0.039773	0.049741	−0.000809	−227.2
aug-cc-pVTZ	−10392.8618642	0.039354	0.048987	0.000296	−195.4
**(*E*)-1 + Br$^{\bullet}$**					
6-31+G* (B, C, H)/SVP (Br)	−10391.2890588	0.053829	0.064107	0.012340	
Def2TZVPP	−10392.8349987	0.052312	0.062500	0.011043	–
aug-cc-pVTZ	−10392.8667630	0.049099	0.059247	0.008116	
**TS^{add}**					
6-31+G* (B, C, H)/SVP (Br)	−10391.2837031	0.047129	0.057201	0.004939	−14.5
Def2TZVPP	−10392.8272627	0.044610	0.054674	0.002367	−23.3
aug-cc-pVTZ	−10392.8565891	0.043752	0.053768	0.002085	−26.6
**^{add}(*E*)-1 + Br$^{\bullet}$**					
6-31+G* (B, C, H)/SVP (Br)	−10391.2849680	0.039204	0.050302	−0.005458	
Def2TZVPP	−10392.8285995	0.039304	0.050230	−0.004262	–
aug-cc-pVTZ	−10392.8583305	0.039330	0.050247	−0.004237	
**Figure 8 B3LYP-GD3BJ**	**E_{el}**	**ZPE corrections**	**H_{corr}**	**G_{corr}**	**Imaginary frequency**
**(*Z*)-1 + Br$^{\bullet}$**					
6-31+G* (B, C, H)/SVP (Br)	−10397.8224825	0.038390	0.049180	−0.003452	
Def2TZVPP	−10399.1041085	0.038244	0.049000	−0.003341	–
aug-cc-pVTZ	−10399.2890225	0.038219	0.048999	−0.003640	
6-31+G* (B, C, H)/ECP28MWB (Br)	−155.841389135	0.038732	0.049438	−0.003067	
**TS3′**					
6-31+G* (B, C, H)/SVP (Br)	-10397.8096540	0.037516	0.047359	-0.002372	-82.0
Def2TZVPP	-10399.0911560	0.037245	0.047089	-0.002537	-80.3
aug-cc-pVTZ	-10399.2757992	0.037195	0.047048	-0.002591	-80.8
6-31+G* (B, C, H)/ECP28MWB (Br)	-155.820838314	0.037326	0.047181	-0.002406	-94.5
**(*E*)-1 + Br$^{\bullet}$**					
6-31+G* (B, C, H)/SVP (Br)	−10397.8270080	0.038575	0.049342	−0.002938	
Def2TZVPP	−10399.1087867	0.038513	0.049244	−0.002868	–
aug-cc-pVTZ	−10399.2937139	0.038397	0.049149	−0.003061	
6-31+G* (B, C, H)/ECP28MWB (Br)	−155.846221404	0.038723	0.049501	−0.002956	
**Figure 9&10 MP2**	**E_{el}**	**ZPE corrections**	**H_{corr}**	**G_{corr}**	**Imaginary frequency**
**(*Z*)-1 + Br$^{\bullet}$**					
6-31+G* (B, C, H)/SVP (Br)	−10391.2829261	0.046368	0.056636	0.004194	
Def2TZVPP	−10392.8292079	0.084352	0.094441	0.042928	–
aug-cc-pVTZ	−10392.8609393	0.063139	0.073188	0.022003	

**TS2′**					
6-31+G* (B, C, H)/SVP (Br)	−10391.2748451	0.039907	0.049862	−0.001010	−163.7
Def2TZVPP	−10392.8223098	0.040036	0.049902	−0.000393	−195.5
aug-cc-pVTZ	−10392.8543104	0.039970	0.049840	−0.000371	−230.7
**IM3**					
6-31+G* (B, C, H)/SVP (Br)	−10391.2828917	0.039452	0.049745	−0.001270	
Def2TZVPP	−10392.8328423	0.038920	0.049276	−0.001820	–
aug-cc-pVTZ	−10392.8654458	0.038803	0.049205	−0.002109	
**TS3′a**					
6-31+G* (B, C, H)/SVP (Br)	−10391.2787973	0.039037	0.048596	−0.000659	-71.9
Def2TZVPP	−10392.8287549	0.038723	0.048259	−0.000645	-56.6
aug-cc-pVTZ	−10392.8614090	0.038623	0.048190	−0.000770	-59.0
**IM3a**					
6-31+G* (B, C, H)/SVP (Br)	−10391.2826159	0.039359	0.049578	−0.001231	
Def2TZVPP	−10392.8322242	0.038893	0.049171	−0.001758	–
aug-cc-pVTZ	−10392.8648914	0.038866	0.049135	−0.001704	
**TS3′b**					
6-31+G* (B, C, H)/SVP (Br)	−103912821022	0.038348	0.048302	−0.002236	-313.1
Def2TZVPP	−103928319952	0.037983	0.047941	−0.002513	-220.3
aug-cc-pVTZ	−103928645431	0.037842	0.047845	−0.002862	-255.8
**IM3b**					
6-31+G* (B, C, H)/SVP (Br)	−10391.2840324	0.039477	0.049719	−0.001893	
Def2TZVPP	−10392.8335411	0.039051	0.049322	−0.002175	–
aug-cc-pVTZ	−10392.8659613	0.038843	0.049184	−0.002952	
**TS3′c**					
6-31+G* (B, C, H)/SVP (Br)	−10391.2837035	0.039228	0.048702	−0.000365	-33.6
Def2TZVPP	−10392.8332905	0.038826	0.048328	−0.000735	-32.3
aug-cc-pVTZ	−10392.8656110	0.038705	0.048240	−0.000963	-35.0
**IM4**					
6-31+G* (B, C, H)/SVP (Br)	−10391.2877791	0.039608	0.049892	−0.001269	
Def2TZVPP	−10392.8374572	0.039269	0.049534	−0.001283	–
aug-cc-pVTZ	−10392.8699920	0.039210	0.049477	−0.001316	
**TS4′**					
6-31+G* (B, C, H)/SVP (Br)	−10391.2811989	0.039903	0.049853	−0.000529	−183.4
Def2TZVPP	−10392.8285547	0.039773	0.049741	−0.000809	−227.2
aug-cc-pVTZ	−10392.8618642	0.039354	0.048987	0.000296	−195.4
**(*E*)-1 + Br•**					
6-31+G* (B, C, H)/SVP (Br)	−10391.2890588	0.053829	0.064107	0.012340	
Def2TZVPP	−10392.8349987	0.052312	0.062500	0.011043	–
aug-cc-pVTZ	−10392.8667630	0.049099	0.059247	0.008116	
**TS^{add}**					
6-31+G* (B, C, H)/SVP (Br)	−10391.2837031	0.047129	0.057201	0.004939	−14.5
Def2TZVPP	−10392.8272627	0.044610	0.054674	0.002367	−23.3
aug-cc-pVTZ	−10392.8565891	0.043752	0.053768	0.002085	−26.6
**^{add}(*E*)-1 + Br•**					
6-31+G* (B, C, H)/SVP (Br)	−10391.2849680	0.039204	0.050302	−0.005458	
Def2TZVPP	−10392.8285995	0.039304	0.050230	−0.004262	–
aug-cc-pVTZ	−10392.8583305	0.039330	0.050247	−0.004237	
**Figure 11 B3LYP-GD3BJ**	**E_{el}**	**ZPE corrections**	**H_{corr}**	**G_{corr}**	**Imaginary frequency**
**(*Z*)-1 + Br•**					
6-31+G* (B, C, H)/SVP (Br)	−10397.8224825	0.038390	0.049180	−0.003452	
Def2TZVPP	−10399.1041085	0.038244	0.049000	−0.003341	–
aug-cc-pVTZ	−10399.2890225	0.038219	0.048999	−0.003640	
**TS3′a**					
6-31+G* (B, C, H)/SVP (Br)	-10397.8133508	0.037996	0.047685	-0.001747	-65.3
Def2TZVPP	-10399.0945490	0.037709	0.047410	-0.002020	-62.8
aug-cc-pVTZ	-10399.2792673	0.037656	0.047367	-0.002086	-61.6

**IM3a**						
6-31+G* (B, C, H)/SVP (Br)	-10397.8135276	0.038071	0.048575	-0.003158		
Def2TZVPP	-10399.0947724	0.037717	0.048268	-0.003609		–
aug-cc-pVTZ	-10399.2794665	0.037656	0.048223	-0.003746		
**TS3'b**						
6-31+G* (B, C, H)/SVP (Br)	-10397.8133374	0.037404	0.047427	-0.003079	-176.9	
Def2TZVPP	-10399.0947270	0.037276	0.047197	-0.002825	-97.5	
aug-cc-pVTZ	-10399.2794248	0.037207	0.047161	-0.002924	-127.7	
**(*E*)-1 + Br•**						
6-31+G* (B, C, H)/SVP (Br)	−10397.8270080	0.038575	0.049342	−0.002938		
Def2TZVPP	−10399.1087867	0.038513	0.049244	−0.002868		–
aug-cc-pVTZ	−10399.2937139	0.038397	0.049149	−0.003061		

6. Optimized Cartesian Coordinates

6.1 B3LYP-GD3BJ cartesian coordinates (in Å) referring to Figure 1. (a)

Table S2: B3LYP-GD3BJ cartesian coordinates (in Å) referring to **Figure 1**. **(a)**

**C_2H_2 + BBr_3**, 6-31+G* (B, C, H)/SVP (Br)	*x*	*y*	*z*
B	−0.229849	0.071091	3.854692
C	−0.363334	−0.191290	0.388401
C	0.794293	0.157574	0.399358
Br	1.600328	0.549418	4.121928
Br	−0.740058	−1.768994	3.843292
Br	−1.555531	1.433158	3.673333
H	−1.387734	−0.500430	0.372510
H	1.819015	0.466483	0.404476
**C_2H_2 + BBr_3**, Def2TZVPP	*x*	*y*	*z*

B	−0.238440	0.070301	3.861920
C	−0.347858	−0.192819	0.374702
C	0.796584	0.160651	0.400365
Br	1.584998	0.559196	4.143401
Br	−0.736888	−1.771831	3.842601
Br	−1.570159	1.423388	3.667067
H	−1.365162	−0.506963	0.350158
H	1.814057	0.475088	0.417776
**C_2H_2 + BBr_3**, aug-cc-pVTZ	*x*	*y*	*z*
B	−0.232958	0.071227	3.858141
C	−0.353833	−0.191381	0.379963
C	0.791530	0.157464	0.402640
Br	1.592649	0.558386	4.133763
Br	−0.733798	−1.771121	3.840989
Br	−1.564457	1.426424	3.668535
H	−1.371877	−0.501681	0.357488
H	1.809875	0.467693	0.416471
**C_2H_2 + BBr_3**, 6-31+G* (B, C, H)/ECP28MWB (Br)	*x*	*y*	*z*
B	−0.223666	0.073298	3.860107
C	−0.368542	−0.195204	0.375933
C	0.787946	0.157063	0.399042
Br	1.617424	0.552816	4.145216
Br	−0.739422	−1.779572	3.843327
Br	−1.556487	1.447180	3.673186
H	−1.391975	−0.507507	0.349861
H	1.811852	0.468936	0.411318
**TS1**, 6-31+G* (B, C, H)/SVP (Br)	*x*	*y*	*z*
B	−0.279170	0.000667	2.951163
C	−0.353676	−0.121366	1.239406
C	0.805215	0.177080	0.881214
Br	1.603971	0.543145	3.568557
Br	−0.748827	−1.826805	3.614195
Br	−1.616433	1.410832	3.422761
H	−1.286342	−0.404962	0.767902
H	1.812402	0.438419	0.612790
**TS1**, Def2TZVPP	*x*	*y*	*z*
B	−0.295838	−0.002934	2.941538
C	−0.348865	−0.119751	1.248069
C	0.807437	0.176491	0.906757
Br	1.605816	0.549025	3.531336
Br	−0.746845	−1.826620	3.626117
Br	−1.622990	1.409905	3.431169
H	−1.267056	−0.399757	0.756199
H	1.805482	0.430652	0.616803
**TS1**, aug-cc-pVTZ	*x*	*y*	*z*
B	−0.297136	−0.002580	2.939688
C	−0.348901	−0.120891	1.248647
C	0.807398	0.175387	0.908114
Br	1.606955	0.551774	3.527457
Br	−0.746005	−1.825714	3.631409
Br	−1.624292	1.411288	3.432476
H	−1.265555	−0.401340	0.754364
H	1.804676	0.429088	0.615833
**TS1**, 6-31+G* (B, C, H)/ECP28MWB (Br)	*x*	*y*	*z*
B	−0.181723	0.049563	3.067704
C	0.214412	−0.601229	1.132338
C	0.222953	0.542165	0.703975
Br	1.452615	−0.705084	3.920035

Br	−1.864393	−0.985595	3.325243
Br	−0.403275	2.018710	3.353651
H	0.278715	−1.672475	1.151222
H	0.217836	1.570956	0.403820
**(Z)-1**, 6-31+G* (B, C, H)/SVP (Br)	*x*	*y*	*z*
B	0.005755	0.005183	−0.000461
C	0.987041	1.184200	0.001582
C	2.331225	1.264380	0.000712
Br	3.592314	−0.144239	−0.003178
Br	0.477087	−1.853517	0.005569
Br	−1.880821	0.433534	−0.009259
H	0.525872	2.174339	0.003213
H	2.838426	2.223620	0.001821
**(Z)-1**, Def2TZVPP	*x*	*y*	*z*
B	0.012048	0.008347	0.001420
C	0.992003	1.180422	0.001565
C	2.326341	1.264478	−0.001136
Br	3.592753	−0.138241	−0.006392
Br	0.471490	−1.853163	0.014293
Br	−1.874336	0.439874	−0.011498
H	0.528136	2.164501	0.003079
H	2.828464	2.221283	−0.001331
**(Z)-1**, aug-cc-pVTZ	*x*	*y*	*z*
B	0.012409	0.008489	0.013530
C	0.992073	1.180677	−0.000367
C	2.325895	1.265362	−0.013308
Br	3.593744	−0.138363	−0.018523
Br	0.471180	−1.853900	0.049859
Br	−1.875216	0.439232	−0.004101
H	0.528484	2.164527	−0.002903
H	2.828332	2.221476	−0.024187
**(Z)-1**, 6-31+G* (B, C, H)/ECP28MWB (Br)	*x*	*y*	*z*
B	0.004217	0.005126	−0.000278
C	0.987873	1.179240	0.003031
C	2.327926	1.275609	0.000532
Br	3.628962	−0.147758	−0.008966
Br	0.463982	−1.872281	0.011273
Br	−1.898376	0.444588	−0.015713
H	0.525285	2.169580	0.007269
H	2.837031	2.233395	0.002851

6.2 MP2 cartesian coordinates (in Å) referring to Figure 1. (b)

Table S3: MP2 cartesian coordinates (in Å) referring to **Figure 1**. **(b)**

**C_2H_2 + BBr_3**, 6-31+G* (B, C, H)/SVP (Br)	*x*	*y*	*z*
B	−0.217286	0.074446	3.901074
C	−0.380240	−0.204430	0.343858
C	0.785885	0.163834	0.358114
Br	1.609820	0.517613	4.179381
Br	−0.757311	−1.746769	3.889855
Br	−1.509694	1.452516	3.706764
H	−1.401456	−0.526149	0.311520
H	1.807411	0.485948	0.367424
**C_2H_2 + BBr_3**, Def2TZVPP	*x*	*y*	*z*
B	−0.230770	0.069245	3.825925
C	−0.367885	−0.176600	0.413720
C	0.801322	0.146799	0.440291
Br	1.566720	0.586294	4.116943

Br	−0.693025	−1.765562	3.803647
Br	−1.573730	1.388209	3.629628
H	−1.393806	−0.462265	0.375925
H	1.828303	0.430900	0.451920
**C_2H_2 + BBr_3**, aug-cc-pVTZ	*x*	*y*	*z*
B	−0.217511	0.072372	3.774805
C	−0.383684	−0.170337	0.461832
C	0.791864	0.133398	0.483573
Br	1.576400	0.599242	4.059966
Br	−0.672924	−1.762510	3.778724
Br	−1.566069	1.383047	3.577789
H	−1.414460	−0.438569	0.428112
H	1.823514	0.400366	0.493189
**TS1**, 6-31+G* (B, C, H)/SVP (Br)	*x*	*y*	*z*
B	−0.352645	−0.024918	2.925982
C	−0.359647	−0.113466	1.273380
C	0.835988	0.195083	1.008034
Br	1.603308	0.525444	3.411313
Br	−0.748636	−1.823846	3.653694
Br	−1.615507	1.395790	3.482911
H	−1.234906	−0.377617	0.686455
H	1.809186	0.440540	0.616219
**TS1**, Def2TZVPP	*x*	*y*	*z*
B	−0.334249	−0.014859	2.914679
C	−0.369891	−0.124214	1.249287
C	0.823532	0.186030	1.037380
Br	1.596838	0.538686	3.389052
Br	−0.725781	−1.802476	3.644918
Br	−1.598582	1.398058	3.450851
H	−1.255718	−0.394330	0.698177
H	1.801002	0.430105	0.673657
**TS1**, aug-cc-pVTZ	*x*	*y*	*z*
B	−0.332791	−0.017391	2.912438
C	−0.372559	−0.120464	1.246600
C	0.822003	0.189157	1.038623
Br	1.596931	0.532403	3.388957
Br	−0.724737	−1.804264	3.638555
Br	−1.593992	1.391360	3.458907
H	−1.259413	−0.387206	0.694395
H	1.801700	0.433417	0.679514
**(Z)-1**, 6-31+G* (B, C, H)/SVP (Br)	*x*	*y*	*z*
B	0.018026	−0.001511	0.025191
C	0.984728	1.191561	0.153548
C	2.322260	1.272278	−0.020578
Br	3.482683	−0.132960	−0.506617
Br	0.444389	−1.798661	0.490170
Br	−1.761063	0.379815	−0.580301
H	0.530320	2.162425	0.366564
H	2.855556	2.214553	0.072021
**(Z)-1**, Def2TZVPP	*x*	*y*	*z*
B	0.009685	0.003134	0.007914
C	0.986161	1.184506	−0.004673
C	2.330997	1.260125	−0.008218
Br	3.562179	−0.142803	0.002805
Br	0.483118	−1.834926	0.011172
Br	−1.856292	0.427035	0.020709
H	0.530761	2.171149	−0.011896
H	2.830291	2.219280	−0.017813

**(Z)-1**, aug-cc-pVTZ	*x*	*y*	*z*
B	0.011269	0.003974	0.000001
C	0.985239	1.186912	0.000003
C	2.330289	1.260390	0.000000
Br	3.553458	−0.148924	−0.000010
Br	0.487079	−1.831496	0.000010
Br	−1.853503	0.424352	−0.000011
H	0.529448	2.174035	0.000007
H	2.833621	2.218247	0.000001

6.3 B3LYP-GD3BJ cartesian coordinates (in Å) referring to Figures 2 and 3

Table S4: B3LYP-GD3BJ cartesian coordinates (in Å) referring to **Figure 2** and **Figure 3**.

**(Z)-1 + Br$^•$**, 6-31+G* (B, C, H)/SVP (Br)	*x*	*y*	*z*
B	0.723372	0.155188	0.309040
C	1.742445	1.336598	0.275750
C	3.098453	1.399254	−0.013999
Br	4.293065	−0.023479	−0.137497
Br	1.174821	−1.710010	0.220808
Br	−1.141143	0.627608	0.084282
H	1.289013	2.326677	0.231583
H	3.581840	2.359164	−0.161757
Br	1.537085	1.071251	2.694010
**(Z)-1 + Br$^•$**, Def2TZVPP	*x*	*y*	*z*
B	−0.926648	0.035242	−0.220681
C	0.117632	1.182492	−0.315160
C	1.454586	1.211848	−0.652564
Br	2.611535	−0.236415	−0.802107
Br	−0.535562	−1.843648	−0.256324
Br	−2.785591	0.549070	−0.374623
H	−0.317823	2.175169	−0.368420

H	1.949249	2.155052	−0.834719
Br	0.015790	1.031561	2.114287
**(Z)-1 + Br**$^{\bullet}$, aug-cc-pVTZ	*x*	*y*	*z*
B	0.724189	0.157858	0.292888
C	1.745440	1.329129	0.278501
C	4.288643	−0.019750	−0.157820
Br	1.158088	−1.710349	0.197574
Br	−1.140414	0.638437	0.093893
Br	−2.845470	0.467880	−0.539580
H	1.291707	2.314333	0.246966
H	3.571387	2.355905	−0.142270
Br	1.567969	1.079188	2.700909
**(Z)-1 + Br**$^{\bullet}$, 6-31+G* (B, C, H)/ECP28MWB (Br)	*x*	*y*	*z*
B	−0.986049	0.012998	−0.278694
C	0.043938	1.175095	−0.347872
C	1.388678	1.231047	−0.605737
Br	2.593400	−0.221499	−0.830196
Br	−0.568265	−1.868814	−0.238900
Br	−2.862829	0.511170	−0.383709
H	−0.391902	2.175655	−0.349097
H	1.899215	2.183346	−0.706876
Br	0.105734	1.092252	2.311551
**TS2**, 6-31+G* (B, C, H)/SVP (Br)	*x*	*y*	*z*
B	0.584741	0.098480	0.032005
C	1.530436	1.296655	0.222264
C	2.920811	1.370742	0.313656
Br	4.100449	−0.000241	−0.245279
Br	0.996741	−1.703549	0.511908
Br	−1.152998	0.482403	−0.704323
H	1.059712	2.276095	0.322896
H	3.410492	2.336642	0.280681
Br	2.848556	1.385043	2.768422
**TS2**, Def2TZVPP	*x*	*y*	*z*
B	0.587507	0.101046	0.028837
C	1.532749	1.290150	0.221604
C	2.915536	1.368605	0.321982
Br	4.104816	0.005914	−0.234616
Br	0.990269	−1.704147	0.504926
Br	−1.152347	0.489840	−0.699842
H	1.060239	2.264429	0.318519
H	3.397573	2.333100	0.287637
Br	2.862598	1.393333	2.753182
**TS2**, aug-cc-pVTZ	*x*	*y*	*z*
B	0.587119	0.100992	0.028364
C	1.531970	1.290195	0.224159
C	2.914539	1.369093	0.322992
Br	4.103759	0.005265	−0.236812
Br	0.987691	−1.704789	0.507677
Br	−1.150606	0.489271	−0.707761
H	1.059247	2.263827	0.322481
H	3.396554	2.333155	0.287153
Br	2.868666	1.395262	2.753978
**TS2**, 6-31+G* (B, C, H)/ECP28MWB (Br)	*x*	*y*	*z*
B	0.575391	0.098266	0.030785
C	1.499081	1.301563	0.270048
C	2.893341	1.393242	0.361718
Br	4.068115	0.029096	−0.354672
Br	0.909443	−1.678603	0.689317

Br	−1.060524	0.434864	−0.959041
H	1.011276	2.268796	0.412355
H	3.371195	2.363700	0.296057
Br	3.031623	1.331347	2.755663
**IM1**, 6-31+G* (B, C, H)/SVP (Br)	*x*	*y*	*z*
B	0.555319	0.134300	0.103084
C	1.513652	1.321134	0.133607
C	2.890703	1.398096	0.657024
Br	4.109009	0.222993	−0.378695
Br	0.986681	−1.625402	0.730065
Br	−1.209520	0.442989	−0.617775
H	1.169818	2.267463	−0.285643
H	3.324486	2.388425	0.567161
Br	2.958802	0.992261	2.593393
**IM1**, Def2TZVPP	*x*	*y*	*z*
B	0.560226	0.137040	0.103221
C	1.515729	1.316384	0.140091
C	2.887475	1.397590	0.654342
Br	4.106672	0.226169	−0.378248
Br	0.977190	−1.625994	0.730882
Br	−1.200209	0.446544	−0.629070
H	1.166915	2.259594	−0.270046
H	3.317866	2.383799	0.560961
Br	2.967086	1.001135	2.590088
**IM1**, aug-cc-pVTZ	*x*	*y*	*z*
B	0.559685	0.137403	0.103758
C	1.516313	1.315968	0.139000
C	2.886701	1.398796	0.655040
Br	4.109220	0.228197	−0.378858
Br	0.975934	−1.627113	0.730895
Br	−1.202556	0.447494	−0.626428
H	1.169510	2.257524	−0.275597
H	3.316542	2.384928	0.562916
Br	2.967601	0.999062	2.591494
**IM1**, 6-31+G* (B, C, H)/ECP28MWB (Br)	*x*	*y*	*z*
B	−1.161967	0.020273	−0.458327
C	−0.184416	1.190502	−0.433861
C	1.165792	1.285170	0.131358
Br	2.445879	0.036624	−0.817480
Br	−0.789091	−1.740350	0.243202
Br	−2.907949	0.333908	−1.263192
H	−0.499679	2.121840	−0.907510
H	1.618122	2.264600	0.017592
Br	1.174637	0.956513	2.122128
**TS3**, 6-31+G* (B, C, H)/SVP (Br)	*x*	*y*	*z*
B	0.294424	0.849600	0.163647
C	1.746562	0.384169	0.161524
C	2.975460	0.924999	0.777904
Br	2.921350	2.798593	1.346246
Br	−0.534168	1.882087	1.550898
Br	−0.813642	0.237088	−1.296901
H	1.933440	−0.566653	−0.339293
H	3.287650	0.354003	1.654420
Br	4.487893	0.678384	−0.516224
**TS3**, Def2TZVPP	*x*	*y*	*z*
B	0.298472	0.847481	0.164762
C	1.747937	0.399435	0.153435
C	2.970450	0.925268	0.775744

Br	2.937411	2.792620	1.359059
Br	−0.531914	1.893258	1.541068
Br	−0.819698	0.201738	−1.274371
H	1.935126	−0.538118	−0.362705
H	3.270086	0.347454	1.647023
Br	4.491100	0.673133	−0.501795
**TS3**, aug-cc-pVTZ	*x*	*y*	*z*
B	0.298347	0.847323	0.164621
C	1.748049	0.399960	0.152950
C	2.969972	0.924471	0.776400
Br	2.938896	2.792558	1.362634
Br	−0.532589	1.896938	1.538998
Br	−0.821824	0.197404	−1.272503
H	1.935846	−0.536131	−0.364755
H	3.270008	0.345677	1.646471
Br	4.492263	0.674070	−0.502595
**TS3**, 6-31+G* (B, C, H)/ECP28MWB (Br)	*x*	*y*	*z*
B	0.290931	0.846130	0.167261
C	1.747960	0.399565	0.146813
C	2.968627	0.921992	0.778282
Br	2.939908	2.837621	1.330191
Br	−0.549900	1.854833	1.585145
Br	−0.836282	0.232750	−1.299797
H	1.932612	−0.543813	−0.369789
H	3.265554	0.362562	1.667210
Br	4.539559	0.630629	−0.503095
**IM2**, 6-31+G* (B, C, H)/SVP (Br)	*x*	*y*	*z*
B	0.330844	0.534728	0.309191
C	1.758318	0.773545	−0.161865
C	2.873583	1.109347	0.731921
Br	3.676852	2.832998	0.192957
Br	−0.160348	0.724051	2.162549
Br	−1.008662	0.021241	−0.969931
H	2.011596	0.681957	−1.216547
H	2.610258	1.221197	1.778480
Br	4.206529	−0.356785	0.675454
**IM2**, Def2TZVPP	*x*	*y*	*z*
B	0.338444	0.529379	0.310396
C	1.757648	0.781258	−0.155517
C	2.868814	1.118977	0.728998
Br	3.692527	2.822307	0.173122
Br	−0.156060	0.688536	2.165356
Br	−1.001276	0.027887	−0.973781
H	2.006148	0.694710	−1.207075
H	2.610384	1.243624	1.770491
Br	4.182341	−0.364398	0.690219
**IM2**, aug-cc-pVTZ	*x*	*y*	*z*
B	0.337761	0.531056	0.310134
C	1.758166	0.778798	−0.154845
C	2.868858	1.116237	0.729481
Br	3.687858	2.825363	0.176653
Br	−0.159911	0.697004	2.164551
Br	−1.002366	0.027622	−0.974313
H	2.006737	0.690608	−1.205879
H	2.612235	1.237787	1.771443
Br	4.189631	−0.362196	0.684984
**IM2**, 6-31+G* (B, C, H)/ECP28MWB (Br)	*x*	*y*	*z*
B	−1.318103	−0.450909	−0.142410

C	0.126883	−0.308376	−0.597940
C	1.238272	0.040633	0.281497
Br	1.849440	1.921674	−0.190326
Br	−1.840265	−0.174456	1.707133
Br	−2.676859	−0.918654	−1.441714
H	0.378028	−0.439320	−1.649035
H	1.019571	0.081938	1.343169
Br	2.745642	−1.267010	0.082777
**TS4**, 6-31+G* (B, C, H)/SVP (Br)	*x*	*y*	*z*
B	0.361055	0.696720	0.298518
C	1.833341	0.450562	−0.079758
C	2.894136	0.685058	0.796029
Br	2.928468	3.066161	0.268189
Br	−0.176835	1.137727	2.087974
Br	−0.977727	0.521051	−1.063802
H	2.077659	0.128939	−1.090403
H	2.740315	0.863631	1.853565
Br	4.618559	−0.007589	0.431909
**TS4**, Def2TZVPP	*x*	*y*	*z*
B	0.362053	0.692572	0.299179
C	1.830527	0.470847	−0.080067
C	2.889367	0.692845	0.789645
Br	2.962633	3.059735	0.287490
Br	−0.180445	1.148026	2.082763
Br	−0.979331	0.474719	−1.054547
H	2.071333	0.154087	−1.088236
H	2.737790	0.866848	1.843593
Br	4.605043	−0.017419	0.422400
**TS4**, aug-cc-pVTZ	*x*	*y*	*z*
B	0.361690	0.691799	0.299229
C	1.830678	0.472406	−0.080348
C	2.889131	0.693177	0.789500
Br	2.964764	3.060960	0.290013
Br	−0.182549	1.147361	2.083206
Br	−0.980430	0.471453	−1.054698
H	2.071379	0.157458	−1.088728
H	2.738523	0.865674	1.843519
Br	4.605784	−0.018027	0.420529
**TS4**, 6-31+G* (B, C, H)/ECP28MWB (Br)	*x*	*y*	*z*
B	0.345736	0.681967	0.298342
C	1.820454	0.484335	−0.085376
C	2.888758	0.728590	0.786907
Br	3.024493	3.071023	0.308922
Br	−0.209167	1.155510	2.090434
Br	−1.004857	0.417360	−1.060563
H	2.064684	0.179943	−1.101599
H	2.745216	0.865908	1.852262
Br	4.623652	−0.042377	0.412891
**(*E*)-1 + Br•**, 6-31+G* (B, C, H)/SVP (Br)	*x*	*y*	*z*
B	0.506348	0.929030	0.321079
C	1.998657	0.634471	−0.025554
C	2.945216	0.356716	0.944462
Br	2.064691	3.064376	−0.290783
Br	−0.084079	1.175427	2.138899
Br	−0.817065	0.722232	−1.060308
H	2.246274	0.393833	−1.056404
H	2.776229	0.501177	2.005814
Br	4.662688	−0.234993	0.525005

**(*E*)-1 + Br$^{\bullet}$**, Def2TZVPP	*x*	*y*	*z*
B	0.094222	0.016473	0.168668
C	0.974154	1.296248	0.193959
C	2.313479	1.285534	−0.111850
Br	0.900616	0.973414	2.621599
Br	0.855986	−1.740248	−0.023247
Br	−1.814258	0.211498	0.047495
H	0.467707	2.252829	0.200726
H	2.895572	0.381106	−0.210811
Br	3.303831	2.847585	−0.327137

**(*E*)-1 + Br$^{\bullet}$**, aug-cc-pVTZ	*x*	*y*	*z*
B	0.505838	0.915111	0.325383
C	1.995268	0.634504	−0.016035
C	2.940387	0.361147	0.942262
Br	2.092494	3.066007	−0.297764
Br	−0.094381	1.173416	2.137096
Br	−0.814089	0.718600	−1.060125
H	2.240232	0.399547	−1.043516
H	2.776162	0.496842	2.000632
Br	4.657051	−0.222903	0.514278

**(*E*)-1 + Br$^{\bullet}$**, 6-31+G* (B, C, H)/ECP28MWB (Br)	*x*	*y*	*z*
B	1.076275	0.270512	0.055783
C	0.405534	0.591123	0.400490
C	1.341142	0.870537	0.554947
Br	0.779390	2.056375	0.460043
Br	1.703548	0.072975	1.765759
Br	2.373563	0.239646	1.489685
H	0.668300	0.753182	1.444045
H	1.177298	0.776723	1.622968
Br	3.100432	1.461197	0.137991

**TS^{add}**, 6-31+G* (B, C, H)/SVP (Br)	*x*	*y*	*z*
B	−0.032729	0.019317	0.165548
C	0.727715	1.348131	0.292502
C	2.047113	1.393431	0.537158
Br	1.669557	1.750537	3.765732
Br	−1.923595	0.063850	−0.181275
Br	0.820853	−1.695620	0.353299
H	0.174542	2.281215	0.201105
H	2.690527	0.526650	0.641562
Br	3.009517	3.020170	0.747030

**TS^{add}**, Def2TZVPP	*x*	*y*	*z*
B	−0.169093	−0.007843	−0.185706
C	0.635363	1.184338	0.338702
C	1.935149	1.098084	0.623715
Br	1.156184	0.772809	3.873233
Br	−2.041140	0.224027	−0.555762
Br	0.606676	−1.743991	−0.478513
H	0.123567	2.126958	0.496393
H	2.536198	0.209205	0.498509
Br	2.946917	2.548451	1.314446

**TS^{add}**, aug-cc-pVTZ	*x*	*y*	*z*
B	−0.028923	0.023236	0.167881
C	0.732623	1.344879	0.295821
C	2.044157	1.396181	0.529978
Br	1.670599	1.733518	3.796877
Br	−1.917103	0.071056	−0.197753
Br	0.812534	−1.696011	0.369926
H	0.179478	2.272036	0.202454

H	2.689132	0.536537	0.635110
Br	3.001003	3.026248	0.722367
**TS[add]**, 6-31+G* (B, C, H)/ECP28MWB (Br)	*x*	*y*	*z*
B	−0.160278	−0.030228	−0.195074
C	0.627523	1.179206	0.327931
C	1.933472	1.108813	0.619351
Br	1.134444	0.781771	3.928800
Br	−2.054892	0.173854	−0.552012
Br	0.645091	−1.767978	−0.508262
H	0.100362	2.120073	0.478918
H	2.565363	0.234425	0.510340
Br	2.938736	2.612102	1.315026
**[add](*E*)-1 + Br•**, 6-31+G* (B, C, H)/SVP (Br)	*x*	*y*	*z*
B	−0.910163	−0.665301	−1.357963
C	−0.140689	0.422987	−0.581723
C	1.176163	0.334461	−0.351514
Br	0.876130	1.078574	3.293067
Br	−2.786444	−0.417259	−1.680298
Br	−0.067860	−2.260063	−2.024141
H	−0.687853	1.287005	−0.209176
H	1.824138	−0.478110	−0.660290
Br	2.129807	1.666557	0.612570
**[add](*E*)-1 + Br•**, Def2TZVPP	*x*	*y*	*z*
B	−0.910318	−0.663775	−1.381432
C	−0.182632	0.466014	−0.634229
C	1.091217	0.370345	−0.261143
Br	1.953676	0.545015	3.364765
Br	−2.751995	−0.414645	−1.861261
Br	−0.056873	−2.317751	−1.855934
H	−0.733425	1.370946	−0.404741
H	1.737788	−0.479025	−0.422485
Br	1.988054	1.774686	0.646569
**[add](*E*)-1 + Br•**, aug-cc-pVTZ	*x*	*y*	*z*
B	−0.951458	−0.657318	−1.433078
C	−0.247297	0.508511	−0.719944
C	0.971693	0.392971	−0.200216
Br	1.828341	0.679151	3.385999
Br	−2.698355	−0.365568	−2.175604
Br	−0.157850	−2.398084	−1.616292
H	−0.765707	1.457292	−0.646656
H	1.582998	−0.496671	−0.200523
Br	1.850865	1.848565	0.646845
**[add](*E*)-1 + Br•**, 6-31+G* (B, C, H)/ECP28MWB (Br)	*x*	*y*	*z*
B	−0.956359	−0.603383	−1.463203
C	−0.251351	0.561479	−0.741955
C	0.981365	0.442586	−0.238099
Br	1.481807	0.755734	3.540634
Br	−2.731576	−0.311470	−2.178888
Br	−0.149157	−2.350667	−1.688359
H	−0.778371	1.509238	−0.645278
H	1.614541	−0.436782	−0.255015
Br	1.875890	1.919034	0.651053

6.4 MP2 cartesian coordinates (in Å) referring to Figures 4 and 5

Table S5: MP2 cartesian coordinates (in Å) referring to **Figure 4** and **Figure 5**.

**(Z)-1 + Br•**, 6-31+G* (B, C, H)/SVP (Br)	*x*	*y*	*z*
B	−1.023560	−0.019709	−0.456360
C	−0.025577	1.158757	−0.363679
C	1.330660	1.202375	−0.518389
Br	2.462447	−0.233041	−0.935555
Br	−0.625609	−1.825638	−0.025184
Br	−2.799050	0.420359	−1.020504
H	−0.461808	2.154012	−0.250300
H	1.864781	2.149473	−0.508888
Br	0.860885	1.253783	2.368549
**(Z)-1 + Br•**, Def2TZVPP	*x*	*y*	*z*
B	−1.007823	−0.011758	−0.438965
C	0.003732	1.152166	−0.449341
C	1.370762	1.186494	−0.486814
Br	2.532894	−0.243613	−0.683564
Br	−0.569819	−1.849076	−0.326658
Br	−2.848953	0.469490	−0.547428
H	−0.422220	2.151525	−0.441664
H	1.886763	2.137596	−0.506408
Br	0.637834	1.267547	2.170532
**(Z)-1 + Br•**, aug-cc-pVTZ	*x*	*y*	*z*
B	−1.005130	−0.006136	−0.430855
C	0.002689	1.160661	−0.455216
C	1.372609	1.191581	−0.482991
Br	2.525316	−0.243700	−0.677420
Br	−0.559112	−1.837835	−0.299335
Br	−2.845470	0.467880	−0.539580

H	−0.422073	2.161303	−0.450152
H	1.891599	2.142104	−0.498841
Br	0.622742	1.224512	2.124080
**TS2**, 6-31+G* (B, C, H)/SVP (Br)	*x*	*y*	*z*
B	0.601327	0.092296	0.051559
C	1.512251	1.319780	0.313971
C	2.877176	1.397701	0.288636
Br	3.980878	0.032726	−0.399900
Br	0.912840	−1.634497	0.764290
Br	−0.942873	0.412231	−1.024310
H	1.019381	2.271387	0.521820
H	3.378333	2.360363	0.278388
Br	2.959626	1.290284	2.707776
**TS2**, Def2TZVPP	*x*	*y*	*z*
B	0.613999	0.101534	0.054367
C	1.563696	1.302819	0.232444
C	2.925690	1.369343	0.276670
Br	4.100843	0.003272	−0.190671
Br	1.052895	−1.693094	0.453478
Br	−1.138775	0.501312	−0.575053
H	1.096331	2.278264	0.320634
H	3.416264	2.331878	0.276921
Br	2.667997	1.346942	2.653441
**TS2**, aug-cc-pVTZ	*x*	*y*	*z*
B	0.617822	0.105499	0.059678
C	1.563634	1.309436	0.233623
C	2.926103	1.372025	0.271276
Br	4.091677	0.000374	−0.196290
Br	1.063032	−1.682342	0.471376
Br	−1.131699	0.494557	−0.577499
H	1.098831	2.286468	0.323989
H	3.421102	2.333536	0.275993
Br	2.648440	1.322717	2.640084
**IM1**, 6-31+G* (B, C, H)/SVP (Br)	*x*	*y*	*z*
B	0.554778	0.130108	0.107021
C	1.515836	1.324932	0.129376
C	2.896794	1.392684	0.661935
Br	4.094895	0.231240	−0.367412
Br	0.992103	−1.616565	0.727455
Br	−1.199888	0.443206	−0.600333
H	1.175427	2.266374	−0.302990
H	3.321104	2.390703	0.572994
Br	2.947899	0.979579	2.574176
**IM1**, Def2TZVPP	*x*	*y*	*z*
B	0.561249	0.138308	0.103990
C	1.514450	1.329725	0.135803
C	2.893978	1.387163	0.658543
Br	4.066159	0.223506	−0.360575
Br	1.007481	−1.594725	0.734525
Br	−1.182005	0.429809	−0.617314
H	1.168497	2.272690	−0.273066
H	3.323961	2.375880	0.566844
Br	2.945179	0.979902	2.553470
**IM1**, aug-cc-pVTZ	*x*	*y*	*z*
B	0.565522	0.141335	0.104794
C	1.513463	1.335868	0.133791
C	2.891122	1.388175	0.657353
Br	4.053744	0.215789	−0.360506

Br	1.020825	−1.586983	0.736159
Br	−1.178115	0.422766	−0.612919
H	1.168312	2.279554	−0.275308
H	3.329837	2.374378	0.569601
Br	2.934239	0.971377	2.549256
**TS3**, 6-31+G* (B, C, H)/SVP (Br)	*x*	*y*	*z*
B	0.294185	0.872460	0.147168
C	1.742197	0.373140	0.205024
C	2.993167	0.913484	0.787562
Br	2.928096	2.720946	1.488982
Br	−0.549675	2.016023	1.414940
Br	−0.773501	0.165078	−1.284353
H	1.910908	−0.609148	−0.238322
H	3.367940	0.280321	1.596088
Br	4.385653	0.809965	−0.614870
**TS3**, Def2TZVPP	*x*	*y*	*z*
B	0.280431	0.818062	0.274497
C	1.748168	0.422119	0.133792
C	3.013096	0.940731	0.694330
Br	2.959485	2.682979	1.508113
Br	−0.464803	1.910308	1.634953
Br	−0.918310	0.044444	−1.000204
H	1.923044	−0.474588	−0.452298
H	3.412022	0.260923	1.444990
Br	4.345837	0.937291	−0.735951
**TS3**, aug-cc-pVTZ	*x*	*y*	*z*
B	0.300885	0.864322	0.153663
C	1.751030	0.391664	0.171096
C	2.985818	0.915772	0.786069
Br	2.919275	2.717396	1.454733
Br	−0.514290	2.007254	1.426635
Br	−0.800311	0.137216	−1.227880
H	1.933522	−0.556283	−0.326906
H	3.316279	0.288755	1.613160
Br	4.406762	0.776171	−0.548350
**IM2**, 6-31+G* (B, C, H)/SVP (Br)	*x*	*y*	*z*
B	0.326541	0.533099	0.312425
C	1.759610	0.776264	−0.163458
C	2.880250	1.110054	0.734591
Br	3.675768	2.811993	0.193514
Br	−0.153769	0.724585	2.154402
Br	−0.997537	0.018808	−0.962182
H	2.012204	0.688463	−1.219315
H	2.603930	1.221836	1.780649
Br	4.191971	−0.342822	0.671585
**IM2**, Def2TZVPP	*x*	*y*	*z*
B	0.329627	0.586685	0.298623
C	1.781660	0.674573	−0.148793
C	2.890936	1.026799	0.745937
Br	3.426969	2.846613	0.316679
Br	−0.161501	0.975969	2.097644
Br	−1.008109	0.090975	−0.957135
H	2.050975	0.518334	−1.186439
H	2.631804	1.035154	1.796983
Br	4.356610	−0.212822	0.538710
**IM2**, aug-cc-pVTZ	*x*	*y*	*z*
B	0.352424	0.491337	0.306749
C	1.755726	0.819530	−0.181799

C	2.856376	1.169168	0.723107
Br	3.783577	2.742656	0.105670
Br	−0.048998	0.513090	2.167853
Br	−1.005215	0.047993	−0.942466
H	2.012517	0.758759	−1.233288
H	2.546997	1.364624	1.742703
Br	4.045566	−0.364878	0.813682
**TS4**, 6-31+G* (B, C, H)/SVP (Br)	*x*	*y*	*z*
B	0.380783	0.721394	0.311386
C	1.853956	0.429962	−0.067961
C	2.889036	0.663688	0.803870
Br	2.807250	2.981286	0.186314
Br	−0.149236	1.089387	2.106632
Br	−0.934459	0.651946	−1.064789
H	2.095644	0.096695	−1.076211
H	2.730246	0.867422	1.858438
Br	4.625750	0.040480	0.444540
**TS4**, Def2TZVPP	*x*	*y*	*z*
B	0.391878	0.728475	0.306015
C	1.860097	0.468363	−0.082004
C	2.881832	0.645343	0.806891
Br	2.801128	2.933097	0.194264
Br	−0.115528	1.131605	2.091923
Br	−0.938869	0.592023	−1.039275
H	2.110039	0.164253	−1.090735
H	2.701701	0.845719	1.853395
Br	4.606691	0.033381	0.461747
**TS4**, aug-cc-pVTZ	*x*	*y*	*z*
B	0.397535	0.729462	0.305062
C	1.863112	0.466154	−0.089213
C	2.880443	0.638590	0.805460
Br	2.775000	2.924344	0.200318
Br	−0.090294	1.141861	2.092607
Br	−0.944113	0.592320	−1.025069
H	2.115058	0.168663	−1.100092
H	2.692706	0.833996	1.852972
Br	4.609522	0.046869	0.460174
**(*E*)-1 + Br$^•$**, 6-31+G* (B, C, H)/SVP (Br)	*x*	*y*	*z*
B	0.029694	0.004570	−0.001370
C	0.871829	1.302418	−0.012451
C	2.236201	1.283077	0.016181
Br	1.437399	1.037287	2.785174
Br	0.816919	−1.734072	−0.079938
Br	−1.871092	0.163836	0.017979
H	0.364278	2.265836	−0.065831
H	2.826317	0.370440	0.043847
Br	3.279764	2.831048	−0.144190
**(*E*)-1 + Br$^•$**, Def2TZVPP	*x*	*y*	*z*
B	0.035888	0.007737	0.012496
C	0.874499	1.303153	−0.009054
C	2.237626	1.282085	0.038279
Br	1.397505	1.057142	2.638796
Br	0.833897	−1.713086	−0.107252
Br	−1.854846	0.152945	0.085650
H	0.376068	2.264447	−0.062298
H	2.807679	0.362868	0.068419
Br	3.282993	2.807148	−0.105635
**(*E*)-1 + Br$^•$**, aug-cc-pVTZ	*x*	*y*	*z*

B	0.037371	0.014218	0.021598
C	0.872382	1.312321	−0.016097
C	2.237349	1.282749	0.040791
Br	1.379902	1.034873	2.590057
Br	0.849672	−1.698458	−0.093225
Br	−1.850759	0.144886	0.108210
H	0.377773	2.276323	−0.072227
H	2.798351	0.356886	0.074565
Br	3.289268	2.800642	−0.094271
**TS^{add}**, 6-31+G* (B, C, H)/SVP (Br)	*x*	*y*	*z*
B	−0.147362	−0.041978	−0.290478
C	0.614583	1.149650	0.321051
C	1.908719	1.068255	0.571554
Br	1.176888	1.064889	4.419395
Br	−2.012860	0.157142	−0.662407
Br	0.685501	−1.719388	−0.694261
H	0.074341	2.066916	0.553224
H	2.521969	0.193578	0.371131
Br	2.908042	2.472973	1.335810
**TS^{add}**, Def2TZVPP	*x*	*y*	*z*
B	−0.151418	−0.018599	−0.243605
C	0.629267	1.163521	0.347717
C	1.926951	1.069108	0.574517
Br	1.100482	0.989051	4.207498
Br	−2.022193	0.172206	−0.541586
Br	0.672034	−1.679903	−0.687472
H	0.110174	2.082056	0.594819
H	2.513897	0.187651	0.353980
Br	2.950627	2.446947	1.319150
**TS^{add}**, aug-cc-pVTZ	*x*	*y*	*z*
B	−0.185867	0.010306	−0.267983
C	0.560611	1.249705	0.245889
C	1.790851	1.135022	0.707237
Br	1.738669	0.553215	4.045756
Br	−1.960750	0.202038	−0.921326
Br	0.594794	−1.726972	−0.263175
H	0.073312	2.217972	0.249699
H	2.340397	0.202472	0.731236
Br	2.777804	2.568279	1.397686
**^{add}(*E*)-1 + Br$^{•}$**, 6-31+G* (B, C, H)/SVP (Br)	*x*	*y*	*z*
B	−0.893982	−0.650361	−1.273941
C	−0.084307	0.493541	−0.638123
C	1.241850	0.381743	−0.413040
Br	0.527264	0.796941	3.299853
Br	−2.788623	−0.452057	−1.435950
Br	−0.086826	−2.267515	−1.904634
H	−0.596833	1.407205	−0.338449
H	1.843262	−0.486692	−0.666940
Br	2.251425	1.746045	0.411755
**^{add}(*E*)-1 + Br$^{•}$**, Def2TZVPP	*x*	*y*	*z*
B	−0.906382	−0.650341	−1.301859
C	−0.138589	0.485141	−0.611481
C	1.175985	0.387736	−0.359698
Br	0.842485	0.784946	3.245795
Br	−2.756875	−0.415167	−1.677478
Br	−0.076033	−2.290750	−1.800272
H	−0.662414	1.387729	−0.319415
H	1.783456	−0.470001	−0.613528

Br	2.151597	1.749557	0.478466
**^{add}(*E*)-1 + Br$^{•}$**, aug-cc-pVTZ	*x*	*y*	*z*
B	−0.899391	−0.645325	−1.286372
C	−0.131582	0.474969	−0.571360
C	1.188545	0.373480	−0.350145
Br	0.752770	0.841130	3.094199
Br	−2.756759	−0.427102	−1.620122
Br	−0.052867	−2.251214	−1.858059
H	−0.655138	1.365024	−0.240590
H	1.794055	−0.474193	−0.642650
Br	2.173597	1.712081	0.515629

6.5 MP2 cartesian coordinates (in Å) referring to Figures 6 and 7

Table S6: MP2 cartesian coordinates (in Å) referring to **Figure 6** and **Figure 7**.

**(*Z*)-1 + Br$^{•}$**, 6-31+G* (B, C, H)/SVP (Br)	*x*	*y*	*z*
B	−1.023560	−0.019709	−0.456360
C	−0.025577	1.158757	−0.363679
C	1.330660	1.202375	−0.518389
Br	2.462447	−0.233041	−0.935555
Br	−0.625609	−1.825638	−0.025184
Br	−2.799050	0.420359	−1.020504
H	−0.461808	2.154012	−0.250300
H	1.864781	2.149473	−0.508888
Br	0.860885	1.253783	2.368549
**(*Z*)-1 + Br$^{•}$**, Def2TZVPP	*x*	*y*	*z*
B	−1.007823	−0.011758	−0.438965
C	0.003732	1.152166	−0.449341
C	1.370762	1.186494	−0.486814
Br	2.532894	−0.243613	−0.683564
Br	−0.569819	−1.849076	−0.326658
Br	−2.848953	0.469490	−0.547428
H	−0.422220	2.151525	−0.441664
H	1.886763	2.137596	−0.506408
Br	0.637834	1.267547	2.170532
**(*Z*)-1 + Br$^{•}$**, aug-cc-pVTZ	*x*	*y*	*z*
B	−1.005130	−0.006136	−0.430855
C	0.002689	1.160661	−0.455216
C	1.372609	1.191581	−0.482991
Br	2.525316	−0.243700	−0.677420
Br	−0.559112	−1.837835	−0.299335
Br	−2.845470	0.467880	−0.539580
H	−0.422073	2.161303	−0.450152
H	1.891599	2.142104	−0.498841
Br	0.622742	1.224512	2.124080
**TS2′**, 6-31+G* (B, C, H)/SVP (Br)	*x*	*y*	*z*
B	-0.944004	0.009845	-0.321068
C	0.081945	1.182657	-0.393964

C	1.412143	1.206073	-0.591552
Br	2.585602	-0.229677	-0.689437
Br	-0.519824	-1.844374	-0.293244
Br	-2.790031	0.508718	-0.447304
H	-0.355725	2.181422	-0.443564
H	1.928743	2.154531	-0.717437
Br	0.184322	1.091135	2.187250
**TS2′**, Def2TZVPP	*x*	*y*	*z*
B	-0.949595	0.010879	-0.343237
C	0.071568	1.177999	-0.448499
C	1.401359	1.200904	-0.556849
Br	2.569815	-0.223003	-0.614623
Br	-0.523914	-1.834946	-0.344952
Br	-2.792469	0.502001	-0.367478
H	-0.361997	2.172560	-0.493068
H	1.917264	2.147897	-0.648998
Br	0.251138	1.106040	2.107385
**TS2′**, aug-cc-pVTZ	*x*	*y*	*z*
B	-0.949491	0.012873	-0.344536
C	0.066731	1.181744	-0.476501
C	1.399686	1.202143	-0.544876
Br	2.562509	-0.225571	-0.557982
Br	-0.519698	-1.829701	-0.384639
Br	-2.791071	0.498968	-0.300093
H	-0.366712	2.176560	-0.534709
H	1.920463	2.147516	-0.635125
Br	0.260753	1.095799	2.068140
**IM3**, 6-31+G* (B, C, H)/SVP (Br)	*x*	*y*	*z*
B	-0.958002	0.020300	-0.297188
C	0.100788	1.169359	-0.057194
C	1.430947	1.179553	-0.655004
Br	2.675338	-0.188558	-0.476994
Br	-0.527566	-1.822240	-0.440246
Br	-2.787085	0.550124	-0.471289
H	-0.350128	2.144644	-0.259352
H	1.844916	2.107208	-1.033980
Br	0.153973	1.099971	1.980935
**IM3**, Def2TZVPP	*x*	*y*	*z*
B	-0.952181	0.032390	-0.284712
C	0.105708	1.178327	-0.082424
C	1.443671	1.181241	-0.645059
Br	2.669374	-0.173227	-0.416549
Br	-0.517353	-1.800222	-0.474578
Br	-2.784023	0.547935	-0.395470
H	-0.342269	2.150602	-0.274742
H	1.847208	2.086531	-1.068036
Br	0.113044	1.056784	1.931260
**IM3**, aug-cc-pVTZ	*x*	*y*	*z*
B	-0.950049	0.034065	-0.282190
C	0.105097	1.183010	-0.094692
C	1.444716	1.182898	-0.648088
Br	2.658930	-0.177164	-0.404774
Br	-0.511041	-1.795048	-0.477372
Br	-2.782514	0.542773	-0.377027
H	-0.343304	2.157230	-0.279964
H	1.856246	2.087750	-1.065863
Br	0.105101	1.044846	1.919661
**TS3′**, 6-31+G* (B, C, H)/SVP (Br)	*x*	*y*	*z*

B	0.829208	0.046847	0.248615
C	0.016208	1.142495	0.412493
C	1.391969	0.804826	0.761200
Br	2.906190	1.629519	0.076176
Br	0.016255	1.241490	1.463484
Br	2.664887	0.289185	0.201263
H	0.535160	1.535944	1.295858
H	1.558072	0.188859	1.639095
Br	0.243831	0.963697	2.536219
**TS3′**, Def2TZVPP	*x*	*y*	*z*
B	0.829134	0.033798	0.237461
C	0.011323	1.135670	0.436109
C	1.398536	0.819262	0.765327
Br	2.878022	1.663711	0.076865
Br	0.032055	1.243905	1.415832
Br	2.676624	0.235329	0.149599
H	0.530728	1.534727	1.309229
H	1.584974	0.143264	1.584988
Br	0.262588	0.958535	2.476737
**TS3′**, aug-cc-pVTZ	*x*	*y*	*z*
B	0.829513	0.032211	0.240220
C	0.010858	1.129418	0.442291
C	1.399269	0.813217	0.765272
Br	2.870375	1.652937	0.057940
Br	0.034856	1.233801	1.421686
Br	2.676467	0.231305	0.137603
H	0.528938	1.530085	1.316124
H	1.588845	0.165603	1.607915
Br	0.264377	0.954951	2.466398
**IM4**, 6-31+G* (B, C, H)/SVP (Br)	*x*	*y*	*z*
B	0.903870	0.075589	0.047749
C	0.052683	1.117415	0.335756
C	1.437958	0.799517	0.628569
Br	2.504010	2.032286	1.528730
Br	0.251312	1.615321	0.958108
Br	2.737642	0.021400	0.452824
H	0.385886	1.800078	1.066004
H	1.981570	0.011528	0.120683
Br	0.114322	2.169025	1.416409
**IM4**, Def2TZVPP	*x*	*y*	*z*
B	0.900292	0.058916	0.056558
C	0.057546	1.104195	0.375741
C	1.447366	0.806826	0.625201
Br	2.515888	2.047879	1.474711
Br	0.234742	1.595465	0.952066
Br	2.743903	0.049269	0.381839
H	0.373823	1.789030	1.099228
H	1.969687	0.001589	0.134567
Br	0.154536	2.115934	1.372361
**IM4**, aug-cc-pVTZ	*x*	*y*	*z*
B	0.900456	0.054735	0.057441
C	0.057165	1.101719	0.387970
C	1.446753	0.803330	0.628474
Br	2.519073	2.053244	1.455960
Br	0.226300	1.586684	0.950834
Br	2.745027	0.051274	0.365725
H	0.372777	1.791102	1.109052
H	1.964012	0.005181	0.135814

Br	0.159254	2.106269	1.364419
**TS4′**, 6-31+G* (B, C, H)/SVP (Br)	*x*	*y*	*z*
B	0.088000	0.013377	0.131830
C	0.943607	1.318260	0.114738
C	2.276260	1.290532	-0.068991
Br	1.035949	0.966540	2.642188
Br	0.866479	-1.725298	-0.044763
Br	-1.811777	0.188704	0.061602
H	0.429855	2.279196	0.104345
H	2.855853	0.371870	-0.104438
Br	3.307094	2.821269	-0.277102
**TS4′**, Def2TZVPP	*x*	*y*	*z*
B	0.086413	0.022540	0.126310
C	0.930140	1.328175	0.047027
C	2.261825	1.296647	-0.040566
Br	1.089222	0.948755	2.557468
Br	0.874076	-1.702515	-0.060905
Br	-1.809483	0.171856	0.145727
H	0.422931	2.285649	0.025841
H	2.823350	0.371915	-0.037452
Br	3.312835	2.801428	-0.204040
**TS4′**, aug-cc-pVTZ	*x*	*y*	*z*
B	0.116821	0.070543	0.279675
C	0.915750	1.356479	-0.135112
C	2.262214	1.336029	0.037880
Br	1.086308	0.688496	2.367541
Br	0.853569	-1.669994	-0.139562
Br	-1.801599	0.202337	0.406211
H	0.416166	2.304515	-0.307611
H	2.811249	0.414524	0.193466
Br	3.330842	2.821521	-0.143078
**(*E*)-1 + Br•**, 6-31+G* (B, C, H)/SVP (Br)	*x*	*y*	*z*
B	0.029694	0.004570	−0.001370
C	0.871829	1.302418	−0.012451
C	2.236201	1.283077	0.016181
Br	1.437399	1.037287	2.785174
Br	0.816919	−1.734072	−0.079938
Br	−1.871092	0.163836	0.017979
H	0.364278	2.265836	−0.065831
H	2.826317	0.370440	0.043847
Br	3.279764	2.831048	−0.144190
**(*E*)-1 + Br•**, Def2TZVPP	*x*	*y*	*z*
B	0.035888	0.007737	0.012496
C	0.874499	1.303153	−0.009054
C	2.237626	1.282085	0.038279
Br	1.397505	1.057142	2.638796
Br	0.833897	−1.713086	−0.107252
Br	−1.854846	0.152945	0.085650
H	0.376068	2.264447	−0.062298
H	2.807679	0.362868	0.068419
Br	3.282993	2.807148	−0.105635
**(*E*)-1 + Br•**, aug-cc-pVTZ	*x*	*y*	*z*
B	0.037371	0.014218	0.021598
C	0.872382	1.312321	−0.016097
C	2.237349	1.282749	0.040791
Br	1.379902	1.034873	2.590057
Br	0.849672	−1.698458	−0.093225
Br	−1.850759	0.144886	0.108210

H	0.377773	2.276323	−0.072227
H	2.798351	0.356886	0.074565
Br	3.289268	2.800642	−0.094271
**TS**^{add}, 6-31+G* (B, C, H)/SVP (Br)	*x*	*y*	*z*
B	−0.147362	−0.041978	−0.290478
C	0.614583	1.149650	0.321051
C	1.908719	1.068255	0.571554
Br	1.176888	1.064889	4.419395
Br	−2.012860	0.157142	−0.662407
Br	0.685501	−1.719388	−0.694261
H	0.074341	2.066916	0.553224
H	2.521969	0.193578	0.371131
Br	2.908042	2.472973	1.335810
**TS**^{add}, Def2TZVPP	*x*	*y*	*z*
B	−0.151418	−0.018599	−0.243605
C	0.629267	1.163521	0.347717
C	1.926951	1.069108	0.574517
Br	1.100482	0.989051	4.207498
Br	−2.022193	0.172206	−0.541586
Br	0.672034	−1.679903	−0.687472
H	0.110174	2.082056	0.594819
H	2.513897	0.187651	0.353980
Br	2.950627	2.446947	1.319150
**TS**^{add}, aug-cc-pVTZ	*x*	*y*	*z*
B	−0.185867	0.010306	−0.267983
C	0.560611	1.249705	0.245889
C	1.790851	1.135022	0.707237
Br	1.738669	0.553215	4.045756
Br	−1.960750	0.202038	−0.921326
Br	0.594794	−1.726972	−0.263175
H	0.073312	2.217972	0.249699
H	2.340397	0.202472	0.731236
Br	2.777804	2.568279	1.397686
^{add}**(*E*)-1 + Br$^{\bullet}$**, 6-31+G* (B, C, H)/SVP (Br)	*x*	*y*	*z*
B	−0.893982	−0.650361	−1.273941
C	−0.084307	0.493541	−0.638123
C	1.241850	0.381743	−0.413040
Br	0.527264	0.796941	3.299853
Br	−2.788623	−0.452057	−1.435950
Br	−0.086826	−2.267515	−1.904634
H	−0.596833	1.407205	−0.338449
H	1.843262	−0.486692	−0.666940
Br	2.251425	1.746045	0.411755
^{add}**(*E*)-1 + Br$^{\bullet}$**, Def2TZVPP	*x*	*y*	*z*
B	−0.906382	−0.650341	−1.301859
C	−0.138589	0.485141	−0.611481
C	1.175985	0.387736	−0.359698
Br	0.842485	0.784946	3.245795
Br	−2.756875	−0.415167	−1.677478
Br	−0.076033	−2.290750	−1.800272
H	−0.662414	1.387729	−0.319415
H	1.783456	−0.470001	−0.613528
Br	2.151597	1.749557	0.478466
^{add}**(*E*)-1 + Br$^{\bullet}$**, aug-cc-pVTZ	*x*	*y*	*z*
B	−0.899391	−0.645325	−1.286372
C	−0.131582	0.474969	−0.571360
C	1.188545	0.373480	−0.350145
Br	0.752770	0.841130	3.094199

Br	−2.756759	−0.427102	−1.620122
Br	−0.052867	−2.251214	−1.858059
H	−0.655138	1.365024	−0.240590
H	1.794055	−0.474193	−0.642650
Br	2.173597	1.712081	0.515629

6.6 B3LYP-GD3BJ cartesian coordinates (in Å) referring to Figure 8

Table S7: B3LYP-GD3BJ cartesian coordinates (in Å) referring to **Figure 8**.

**(Z)-1 + Br•**, 6-31+G* (B, C, H)/SVP (Br)	*x*	*y*	*z*
B	0.723372	0.155188	0.309040
C	1.742445	1.336598	0.275750
C	3.098453	1.399254	−0.013999
Br	4.293065	−0.023479	−0.137497
Br	1.174821	−1.710010	0.220808
Br	−1.141143	0.627608	0.084282
H	1.289013	2.326677	0.231583
H	3.581840	2.359164	−0.161757
Br	1.537085	1.071251	2.694010
**(Z)-1 + Br•**, Def2TZVPP	*x*	*y*	*z*
B	−0.926648	0.035242	−0.220681
C	0.117632	1.182492	−0.315160
C	1.454586	1.211848	−0.652564
Br	2.611535	−0.236415	−0.802107
Br	−0.535562	−1.843648	−0.256324
Br	−2.785591	0.549070	−0.374623
H	−0.317823	2.175169	−0.368420
H	1.949249	2.155052	−0.834719
Br	0.015790	1.031561	2.114287
**(Z)-1 + Br•**, aug-cc-pVTZ	*x*	*y*	*z*
B	0.724189	0.157858	0.292888
C	1.745440	1.329129	0.278501
C	4.288643	−0.019750	−0.157820
Br	1.158088	−1.710349	0.197574
Br	−1.140414	0.638437	0.093893
Br	−2.845470	0.467880	−0.539580
H	1.291707	2.314333	0.246966
H	3.571387	2.355905	−0.142270
Br	1.567969	1.079188	2.700909
**(Z)-1 + Br•**, 6-31+G* (B, C, H)/ECP28MWB (Br)	*x*	*y*	*z*
B	−0.986049	0.012998	−0.278694
C	0.043938	1.175095	−0.347872
C	1.388678	1.231047	−0.605737
Br	2.593400	−0.221499	−0.830196
Br	−0.568265	−1.868814	−0.238900
Br	−2.862829	0.511170	−0.383709
H	−0.391902	2.175655	−0.349097
H	1.899215	2.183346	−0.706876
Br	0.105734	1.092252	2.311551

**TS3′**, 6-31+G* (B, C, H)/SVP (Br)	*x*	*y*	*z*
B	0.833202	0.046710	0.230600
C	0.012003	1.145241	0.411885
C	1.395046	0.811603	0.754173
Br	2.917320	1.710754	0.161978
Br	0.005718	1.276404	1.433024
Br	2.680899	0.279556	0.223345
H	0.533193	1.570132	1.277614
H	1.568348	0.091298	1.546697
Br	0.243945	1.001778	2.533981

**TS3′**, Def2TZVPP	*x*	*y*	*z*
B	0.830433	0.040242	0.227778
C	0.008252	1.140149	0.419762
C	1.395256	0.822020	0.760692
Br	2.911017	1.711909	0.154868
Br	0.013574	1.281415	1.415477
Br	2.685414	0.259050	0.200914
H	0.529734	1.561039	1.281428
H	1.574812	0.085464	1.530070
Br	0.257636	0.994188	2.520467

**TS3′**, aug-cc-pVTZ	*x*	*y*	*z*
B	0.830497	0.040130	0.228502
C	0.007894	1.138785	0.420883
C	1.395642	0.820848	0.760764
Br	2.912131	1.710449	0.152040
Br	0.012553	1.281872	1.418114
Br	2.686953	0.259118	0.198739
H	0.528704	1.559690	1.282486
H	1.575863	0.090551	1.535420
Br	0.258952	0.993426	2.521137

**TS3′**, 6-31+G* (B, C, H)/ECP28MWB (Br)	*x*	*y*	*z*
B	0.835853	0.047186	0.234036
C	0.009341	1.127101	0.425500
C	1.397106	0.791361	0.750025
Br	2.950640	1.712334	0.148015
Br	0.001460	1.285262	1.452080
Br	2.703869	0.272104	0.205184
H	0.522156	1.556415	1.293803
H	1.578367	0.125932	1.588088
Br	0.273164	1.014209	2.551749

**(*E*)-1 + Br•**, 6-31+G* (B, C, H)/SVP (Br)	*x*	*y*	*z*
B	0.506348	0.929030	0.321079
C	1.998657	0.634471	−0.025554
C	2.945216	0.356716	0.944462
Br	2.064691	3.064376	−0.290783
Br	−0.084079	1.175427	2.138899
Br	−0.817065	0.722232	−1.060308
H	2.246274	0.393833	−1.056404
H	2.776229	0.501177	2.005814
Br	4.662688	−0.234993	0.525005

**(*E*)-1 + Br•**, Def2TZVPP	*x*	*y*	*z*
B	0.094222	0.016473	0.168668
C	0.974154	1.296248	0.193959
C	2.313479	1.285534	−0.111850
Br	0.900616	0.973414	2.621599
Br	0.855986	−1.740248	−0.023247
Br	−1.814258	0.211498	0.047495
H	0.467707	2.252829	0.200726

H	2.895572	0.381106	−0.210811
Br	3.303831	2.847585	−0.327137
**(*E*)-1 + Br•**, aug-cc-pVTZ	*x*	*y*	*z*
B	0.505838	0.915111	0.325383
C	1.995268	0.634504	−0.016035
C	2.940387	0.361147	0.942262
Br	2.092494	3.066007	−0.297764
Br	−0.094381	1.173416	2.137096
Br	−0.814089	0.718600	−1.060125
H	2.240232	0.399547	−1.043516
H	2.776162	0.496842	2.000632
Br	4.657051	−0.222903	0.514278
**(*E*)-1 + Br•**, 6-31+G* (B, C, H)/ECP28MWB (Br)	*x*	*y*	*z*
B	1.076275	0.270512	0.055783
C	0.405534	0.591123	0.400490
C	1.341142	0.870537	0.554947
Br	0.779390	2.056375	0.460043
Br	1.703548	0.072975	1.765759
Br	2.373563	0.239646	1.489685
H	0.668300	0.753182	1.444045
H	1.177298	0.776723	1.622968
Br	3.100432	1.461197	0.137991

6.7 MP2 Cartesian coordinates (in Å) referring to Figures 9 and 10

Table S8: MP2 Cartesian coordinates (in Å) referring to **Figure 9** and **Figure 10**.

**(Z)-1 + Br•**, 6-31+G* (B, C, H)/SVP (Br)	*x*	*y*	*z*
B	−1.023560	−0.019709	−0.456360
C	−0.025577	1.158757	−0.363679
C	1.330660	1.202375	−0.518389
Br	2.462447	−0.233041	−0.935555
Br	−0.625609	−1.825638	−0.025184
Br	−2.799050	0.420359	−1.020504
H	−0.461808	2.154012	−0.250300
H	1.864781	2.149473	−0.508888
Br	0.860885	1.253783	2.368549
**(Z)-1 + Br•**, Def2TZVPP	*x*	*y*	*z*
B	−1.007823	−0.011758	−0.438965
C	0.003732	1.152166	−0.449341
C	1.370762	1.186494	−0.486814
Br	2.532894	−0.243613	−0.683564
Br	−0.569819	−1.849076	−0.326658
Br	−2.848953	0.469490	−0.547428
H	−0.422220	2.151525	−0.441664
H	1.886763	2.137596	−0.506408
Br	0.637834	1.267547	2.170532
**(Z)-1 + Br•**, aug-cc-pVTZ	*x*	*y*	*z*
B	−1.005130	−0.006136	−0.430855
C	0.002689	1.160661	−0.455216
C	1.372609	1.191581	−0.482991
Br	2.525316	−0.243700	−0.677420
Br	−0.559112	−1.837835	−0.299335
Br	−2.845470	0.467880	−0.539580
H	−0.422073	2.161303	−0.450152
H	1.891599	2.142104	−0.498841
Br	0.622742	1.224512	2.124080
**TS2′**, 6-31+G* (B, C, H)/SVP (Br)	*x*	*y*	*z*
B	−0.944004	0.009845	−0.321068
C	0.081945	1.182657	−0.393964
C	1.412143	1.206073	−0.591552
Br	2.585602	−0.229677	−0.689437
Br	−0.519824	−1.844374	−0.293244
Br	−2.790031	0.508718	−0.447304
H	−0.355725	2.181422	−0.443564
H	1.928743	2.154531	−0.717437
Br	0.184322	1.091135	2.187250
**TS2′**, Def2TZVPP	*x*	*y*	*z*
B	−0.949595	0.010879	−0.343237
C	0.071568	1.177999	−0.448499

C	1.401359	1.200904	−0.556849
Br	2.569815	−0.223003	−0.614623
Br	−0.523914	−1.834946	−0.344952
Br	−2.792469	0.502001	−0.367478
H	−0.361997	2.172560	−0.493068
H	1.917264	2.147897	−0.648998
Br	0.251138	1.106040	2.107385
**TS2′**, aug-cc-pVTZ	*x*	*y*	*z*
B	−0.949491	0.012873	−0.344536
C	0.066731	1.181744	−0.476501
C	1.399686	1.202143	−0.544876
Br	2.562509	−0.225571	−0.557982
Br	−0.519698	−1.829701	−0.384639
Br	−2.791071	0.498968	−0.300093
H	−0.366712	2.176560	−0.534709
H	1.920463	2.147516	−0.635125
Br	0.260753	1.095799	2.068140
**IM3**, 6-31+G* (B, C, H)/SVP (Br)	*x*	*y*	*z*
B	−0.958002	0.020300	−0.297188
C	0.100788	1.169359	−0.057194
C	1.430947	1.179553	−0.655004
Br	2.675338	−0.188558	−0.476994
Br	−0.527566	−1.822240	−0.440246
Br	−2.787085	0.550124	−0.471289
H	−0.350128	2.144644	−0.259352
H	1.844916	2.107208	−1.033980
Br	0.153973	1.099971	1.980935
**IM3**, Def2TZVPP	*x*	*y*	*z*
B	−0.952181	0.032390	−0.284712
C	0.105708	1.178327	−0.082424
C	1.443671	1.181241	−0.645059
Br	2.669374	−0.173227	−0.416549
Br	−0.517353	−1.800222	−0.474578
Br	−2.784023	0.547935	−0.395470
H	−0.342269	2.150602	−0.274742
H	1.847208	2.086531	−1.068036
Br	0.113044	1.056784	1.931260
**IM3**, aug-cc-pVTZ	*x*	*y*	*z*
B	−0.950049	0.034065	−0.282190
C	0.105097	1.183010	−0.094692
C	1.444716	1.182898	−0.648088
Br	2.658930	−0.177164	−0.404774
Br	−0.511041	−1.795048	−0.477372
Br	−2.782514	0.542773	−0.377027
H	−0.343304	2.157230	−0.279964
H	1.856246	2.087750	−1.065863
Br	0.105101	1.044846	1.919661
**TS3′a**, 6-31+G* (B, C, H)/SVP (Br)	*x*	*y*	*z*
B	−0.893239	−0.027692	−0.247612
C	0.090297	1.191601	−0.016990
C	1.483970	1.180251	−0.480085
Br	2.201828	−0.138573	−1.577316
Br	−0.461723	−1.817730	0.215022
Br	−2.614589	0.357608	−0.970338
H	−0.381202	2.133632	−0.316627
H	2.127961	2.036845	−0.316369
Br	0.029888	1.344398	2.000026
**TS3′a**, Def2TZVPP	*x*	*y*	*z*

	x	y	z
B	0.896893	0.013166	0.236507
C	0.098626	1.193747	0.075299
C	1.501691	1.175588	0.478871
Br	2.258995	0.143517	1.517671
Br	0.428566	1.810986	0.136204
Br	2.672598	0.376236	0.795355
H	0.361726	2.132392	0.382161
H	2.137115	2.024224	0.282887
Br	0.053452	1.922256	1.325822
**TS3′a**, aug-cc-pVTZ	*x*	*y*	*z*
B	0.896057	0.010264	0.238518
C	0.098733	1.196495	0.083080
C	1.501075	1.177834	0.481717
Br	2.259349	0.152325	1.501922
Br	0.423117	1.805660	0.129507
Br	2.674321	0.376515	0.782486
H	0.361855	2.138255	0.382053
H	2.137097	2.027042	0.285951
Br	0.057713	1.915931	1.312448
**IM3a**, 6-31+G* (B, C, H)/SVP (Br)	*x*	*y*	*z*
B	−0.872713	−0.047543	−0.193420
C	0.084362	1.207670	−0.042087
C	1.526127	0.977608	−0.314147
Br	1.913649	0.382454	−2.052178
Br	−0.293917	−1.777672	0.335746
Br	−2.627574	0.205160	−0.882590
H	−0.295938	2.081642	−0.588299
H	2.311493	1.558183	0.158973
Br	−0.162288	1.672837	1.867692
**IM3a**, Def2TZVPP	*x*	*y*	*z*
B	−0.872039	−0.034066	−0.186816
C	0.085687	1.214357	−0.072321
C	1.523122	0.985665	−0.321921
Br	1.944907	0.388818	−2.028466
Br	−0.260816	−1.759595	0.296487
Br	−2.652190	0.213953	−0.788614
H	−0.295711	2.076775	−0.624153
H	2.305485	1.513296	0.200589
Br	−0.195244	1.661138	1.814904
**IM3a**, aug-cc-pVTZ	*x*	*y*	*z*
B	−0.870587	−0.030854	−0.190991
C	0.086220	1.216322	−0.073298
C	1.522795	0.984176	−0.316471
Br	1.935641	0.367749	−2.017040
Br	−0.252687	−1.753287	0.285449
Br	−2.650621	0.215330	−0.784409
H	−0.291752	2.083486	−0.621367
H	2.304605	1.526860	0.193323
Br	−0.200414	1.650559	1.814496
**TS3′b**, 6-31+G* (B, C, H)/SVP (Br)	*x*	*y*	*z*
B	−0.871650	−0.043615	−0.161959
C	0.065912	1.235544	−0.085228
C	1.505528	1.003770	−0.354694
Br	1.956055	0.606349	−2.123105
Br	−0.224696	−1.745639	0.379539
Br	−2.653047	0.148532	−0.801015
H	−0.333036	2.061970	−0.687454
H	2.321796	1.187896	0.331526

Br	−0.183662	1.805554	1.792080
**TS3'b**, Def2TZVPP	*x*	*y*	*z*
B	−0.869352	−0.031755	−0.163769
C	0.072138	1.235716	−0.104348
C	1.508467	1.010124	−0.352586
Br	1.976339	0.562631	−2.085167
Br	−0.207546	−1.735615	0.328694
Br	−2.667795	0.171436	−0.726594
H	−0.325178	2.058862	−0.701568
H	2.306204	1.223022	0.337162
Br	−0.210076	1.765929	1.757876
**TS3'b**, aug-cc-pVTZ	*x*	*y*	*z*
B	−0.867020	−0.028099	−0.165701
C	0.071432	1.240537	−0.110165
C	1.507441	1.016568	−0.352004
Br	1.973089	0.553197	−2.078731
Br	−0.193214	−1.727744	0.315934
Br	−2.668778	0.170159	−0.709727
H	−0.325629	2.064251	−0.708266
H	2.303375	1.207620	0.347112
Br	−0.217497	1.763871	1.751236
**IM3b**, 6-31+G* (B, C, H)/SVP (Br)	*x*	*y*	*z*
B	−0.835488	−0.031536	−0.115501
C	0.058318	1.279480	−0.118959
C	1.508486	1.101369	−0.363722
Br	2.011305	0.908353	−2.158255
Br	−0.122897	−1.685385	0.492090
Br	−2.624536	0.063946	−0.751555
H	−0.358662	2.050140	−0.774804
H	2.165889	0.622457	0.353496
Br	−0.219224	1.951547	1.726910
**IM3b**, Def2TZVPP	*x*	*y*	*z*
B	−0.839206	−0.022289	−0.105806
C	0.064380	1.271660	−0.150156
C	1.509415	1.086969	−0.365279
Br	2.059774	0.947968	−2.130385
Br	−0.113833	−1.674949	0.469164
Br	−2.648612	0.085773	−0.659079
H	−0.345734	2.032932	−0.810893
H	2.147888	0.613952	0.363714
Br	−0.250883	1.918355	1.678420
**IM3b**, aug-cc-pVTZ	*x*	*y*	*z*
B	−0.834505	−0.017214	−0.113962
C	0.064628	1.278941	−0.151561
C	1.510124	1.097522	−0.360999
Br	2.042129	0.900462	−2.125964
Br	−0.098163	−1.667684	0.445519
Br	−2.644809	0.087286	−0.654527
H	−0.346879	2.046864	−0.805246
H	2.146714	0.630631	0.375232
Br	−0.256049	1.903563	1.681207
**TS3'c**, 6-31+G* (B, C, H)/SVP (Br)	*x*	*y*	*z*
B	−0.862662	−0.050040	−0.058599
C	0.062319	1.231344	−0.165127
C	1.501494	1.015752	−0.395569
Br	2.164817	1.282105	−2.117030
Br	−0.191603	−1.684074	0.645564
Br	−2.656340	0.053251	−0.685044

H	−0.346479	1.974083	−0.855303
H	2.124992	0.424795	0.264484
Br	−0.213348	2.013134	1.656303
**TS3’c**, Def2TZVPP	*x*	*y*	*z*
B	−0.862665	−0.038217	−0.055609
C	0.067369	1.227608	−0.191519
C	1.502932	1.014809	−0.398187
Br	2.190791	1.273075	−2.092133
Br	−0.174805	−1.673297	0.611957
Br	−2.672946	0.076965	−0.608763
H	−0.338022	1.966167	−0.878815
H	2.114564	0.447271	0.283285
Br	−0.244026	1.965969	1.619463
**TS3’c**, aug-cc-pVTZ	*x*	*y*	*z*
B	0.862965	−0.035201	−0.050899
C	0.068324	1.225722	−0.204164
C	1.503068	1.011603	−0.399180
Br	2.205711	1.301911	−2.079443
Br	−0.169906	−1.662981	0.623714
Br	−2.674569	0.075656	−0.590841
H	−0.336623	1.965898	−0.891002
H	2.101278	0.417097	0.272825
Br	−0.251128	1.960645	1.608670
**IM4**, 6-31+G* (B, C, H)/SVP (Br)	*x*	*y*	*z*
B	0.903870	0.075589	0.047749
C	0.052683	1.117415	0.335756
C	1.437958	0.799517	0.628569
Br	2.504010	2.032286	1.528730
Br	0.251312	1.615321	0.958108
Br	2.737642	0.021400	0.452824
H	0.385886	1.800078	1.066004
H	1.981570	0.011528	0.120683
Br	0.114322	2.169025	1.416409
**IM4**, Def2TZVPP	*x*	*y*	*z*
B	0.900292	0.058916	0.056558
C	0.057546	1.104195	0.375741
C	1.447366	0.806826	0.625201
Br	2.515888	2.047879	1.474711
Br	0.234742	1.595465	0.952066
Br	2.743903	0.049269	0.381839
H	0.373823	1.789030	1.099228
H	1.969687	0.001589	0.134567
Br	0.154536	2.115934	1.372361
**IM4**, aug-cc-pVTZ	*x*	*y*	*z*
B	0.900456	0.054735	0.057441
C	0.057165	1.101719	0.387970
C	1.446753	0.803330	0.628474
Br	2.519073	2.053244	1.455960
Br	0.226300	1.586684	0.950834
Br	2.745027	0.051274	0.365725
H	0.372777	1.791102	1.109052
H	1.964012	0.005181	0.135814
Br	0.159254	2.106269	1.364419
**TS4’**, 6-31+G* (B, C, H)/SVP (Br)	*x*	*y*	*z*
B	0.088000	0.013377	0.131830
C	0.943607	1.318260	0.114738
C	2.276260	1.290532	−0.068991
Br	1.035949	0.966540	2.642188

Br	0.866479	−1.725298	−0.044763
Br	−1.811777	0.188704	0.061602
H	0.429855	2.279196	0.104345
H	2.855853	0.371870	−0.104438
Br	3.307094	2.821269	−0.277102

**TS4′**, Def2TZVPP	*x*	*y*	*z*
B	0.086413	0.022540	0.126310
C	0.930140	1.328175	0.047027
C	2.261825	1.296647	−0.040566
Br	1.089222	0.948755	2.557468
Br	0.874076	−1.702515	−0.060905
Br	−1.809483	0.171856	0.145727
H	0.422931	2.285649	0.025841
H	2.823350	0.371915	−0.037452
Br	3.312835	2.801428	−0.204040

**TS4′**, aug-cc-pVTZ	*x*	*y*	*z*
B	0.116821	0.070543	0.279675
C	0.915750	1.356479	−0.135112
C	2.262214	1.336029	0.037880
Br	1.086308	0.688496	2.367541
Br	0.853569	−1.669994	−0.139562
Br	−1.801599	0.202337	0.406211
H	0.416166	2.304515	−0.307611
H	2.811249	0.414524	0.193466
Br	3.330842	2.821521	−0.143078

**(*E*)-1 + Br$^{\bullet}$**, 6-31+G* (B, C, H)/SVP (Br)	*x*	*y*	*z*
B	0.029694	0.004570	−0.001370
C	0.871829	1.302418	−0.012451
C	2.236201	1.283077	0.016181
Br	1.437399	1.037287	2.785174
Br	0.816919	−1.734072	−0.079938
Br	−1.871092	0.163836	0.017979
H	0.364278	2.265836	−0.065831
H	2.826317	0.370440	0.043847
Br	3.279764	2.831048	−0.144190

**(*E*)-1 + Br$^{\bullet}$**, Def2TZVPP	*x*	*y*	*z*
B	0.035888	0.007737	0.012496
C	0.874499	1.303153	−0.009054
C	2.237626	1.282085	0.038279
Br	1.397505	1.057142	2.638796
Br	0.833897	−1.713086	−0.107252
Br	−1.854846	0.152945	0.085650
H	0.376068	2.264447	−0.062298
H	2.807679	0.362868	0.068419
Br	3.282993	2.807148	−0.105635

**(*E*)-1 + Br$^{\bullet}$**, aug-cc-pVTZ	*x*	*y*	*z*
B	0.037371	0.014218	0.021598
C	0.872382	1.312321	−0.016097
C	2.237349	1.282749	0.040791
Br	1.379902	1.034873	2.590057
Br	0.849672	−1.698458	−0.093225
Br	−1.850759	0.144886	0.108210
H	0.377773	2.276323	−0.072227
H	2.798351	0.356886	0.074565
Br	3.289268	2.800642	−0.094271

**TS^{add}**, 6-31+G* (B, C, H)/SVP (Br)	*x*	*y*	*z*
B	−0.147362	−0.041978	−0.290478
C	0.614583	1.149650	0.321051

C	1.908719	1.068255	0.571554
Br	1.176888	1.064889	4.419395
Br	−2.012860	0.157142	−0.662407
Br	0.685501	−1.719388	−0.694261
H	0.074341	2.066916	0.553224
H	2.521969	0.193578	0.371131
Br	2.908042	2.472973	1.335810
**TS^{add}**, Def2TZVPP	*x*	*y*	*z*
B	−0.151418	−0.018599	−0.243605
C	0.629267	1.163521	0.347717
C	1.926951	1.069108	0.574517
Br	1.100482	0.989051	4.207498
Br	−2.022193	0.172206	−0.541586
Br	0.672034	−1.679903	−0.687472
H	0.110174	2.082056	0.594819
H	2.513897	0.187651	0.353980
Br	2.950627	2.446947	1.319150
**TS^{add}**, aug-cc-pVTZ	*x*	*y*	*z*
B	−0.185867	0.010306	−0.267983
C	0.560611	1.249705	0.245889
C	1.790851	1.135022	0.707237
Br	1.738669	0.553215	4.045756
Br	−1.960750	0.202038	−0.921326
Br	0.594794	−1.726972	−0.263175
H	0.073312	2.217972	0.249699
H	2.340397	0.202472	0.731236
Br	2.777804	2.568279	1.397686
**^{add}(*E*)-1 + Br$^{\bullet}$**, 6-31+G* (B, C, H)/SVP (Br)	*x*	*y*	*z*
B	−0.893982	−0.650361	−1.273941
C	−0.084307	0.493541	−0.638123
C	1.241850	0.381743	−0.413040
Br	0.527264	0.796941	3.299853
Br	−2.788623	−0.452057	−1.435950
Br	−0.086826	−2.267515	−1.904634
H	−0.596833	1.407205	−0.338449
H	1.843262	−0.486692	−0.666940
Br	2.251425	1.746045	0.411755
**^{add}(*E*)-1 + Br$^{\bullet}$**, Def2TZVPP	*x*	*y*	*z*
B	−0.906382	−0.650341	−1.301859
C	−0.138589	0.485141	−0.611481
C	1.175985	0.387736	−0.359698
Br	0.842485	0.784946	3.245795
Br	−2.756875	−0.415167	−1.677478
Br	−0.076033	−2.290750	−1.800272
H	−0.662414	1.387729	−0.319415
H	1.783456	−0.470001	−0.613528
Br	2.151597	1.749557	0.478466
**^{add}(*E*)-1 + Br$^{\bullet}$**, aug-cc-pVTZ	*x*	*y*	*z*
B	−0.899391	−0.645325	−1.286372
C	−0.131582	0.474969	−0.571360
C	1.188545	0.373480	−0.350145
Br	0.752770	0.841130	3.094199
Br	−2.756759	−0.427102	−1.620122
Br	−0.052867	−2.251214	−1.858059
H	−0.655138	1.365024	−0.240590
H	1.794055	−0.474193	−0.642650
Br	2.173597	1.712081	0.515629

6.8 B3LYP-GD3BJ Cartesian coordinates (in Å) referring to Figure 11

Table S9: B3LYP-GD3BJ cartesian coordinates (in Å) referring to **Figure 11**.

**(Z)-1 + Br•**, 6-31+G* (B, C, H)/SVP (Br)	*x*	*y*	*z*
B	0.723372	0.155188	0.309040
C	1.742445	1.336598	0.275750
C	3.098453	1.399254	−0.013999
Br	4.293065	−0.023479	−0.137497
Br	1.174821	−1.710010	0.220808
Br	−1.141143	0.627608	0.084282
H	1.289013	2.326677	0.231583
H	3.581840	2.359164	−0.161757
Br	1.537085	1.071251	2.694010
**(Z)-1 + Br•**, Def2TZVPP	*x*	*y*	*z*
B	−0.926648	0.035242	−0.220681
C	0.117632	1.182492	−0.315160
C	1.454586	1.211848	−0.652564
Br	2.611535	−0.236415	−0.802107
Br	−0.535562	−1.843648	−0.256324
Br	−2.785591	0.549070	−0.374623
H	−0.317823	2.175169	−0.368420
H	1.949249	2.155052	−0.834719
Br	0.015790	1.031561	2.114287
**(Z)-1 + Br•**, aug-cc-pVTZ	*x*	*y*	*z*
B	0.724189	0.157858	0.292888
C	1.745440	1.329129	0.278501
C	4.288643	−0.019750	−0.157820
Br	1.158088	−1.710349	0.197574
Br	−1.140414	0.638437	0.093893
Br	−2.845470	0.467880	−0.539580
H	1.291707	2.314333	0.246966
H	3.571387	2.355905	−0.142270
Br	1.567969	1.079188	2.700909
**TS3'a**, 6-31+G* (B, C, H)/SVP (Br)	*x*	*y*	*z*
B	−0.646411	−0.265880	−0.035475
C	0.303651	0.985841	0.132919
C	1.727569	0.909514	−0.219845
Br	2.279076	−0.146628	−1.672564
Br	−0.113738	−2.031388	0.465987
Br	−2.407104	0.016530	−0.736471
H	−0.154913	1.905594	−0.245107
H	2.428142	1.688302	0.058877
Br	0.125839	1.285004	2.152808
**TS3'a**, Def2TZVPP	*x*	*y*	*z*
B	−0.646289	−0.259959	−0.028697
C	0.307612	0.982731	0.116019
C	1.727634	0.907065	−0.220608
Br	2.293170	−0.128904	−1.675752
Br	−0.105931	−2.028284	0.457206
Br	−2.421009	0.021710	−0.694246
H	−0.147505	1.896089	−0.268696
H	2.429647	1.666469	0.088209
Br	0.104781	1.289973	2.127694
**TS3'a**, aug-cc-pVTZ	*x*	*y*	*z*
B	−0.646150	−0.259611	−0.028125

C	0.307978	0.983082	0.113766
C	1.728712	0.905464	−0.217966
Br	2.292645	−0.125655	−1.679219
Br	−0.104369	−2.028955	0.456843
Br	−2.423508	0.020607	−0.690385
H	−0.145845	1.896260	−0.272012
H	2.431533	1.663211	0.091774
Br	0.101114	1.292486	2.126454
**IM3a**, 6-31+G* (B, C, H)/SVP (Br)	*x*	*y*	*z*
B	−0.646186	−0.271771	−0.009598
C	0.297360	0.993236	0.122137
C	1.739867	0.815536	−0.158663
Br	2.173426	0.115972	−1.855667
Br	−0.048662	−2.010499	0.510460
Br	−2.423180	−0.040105	−0.682266
H	−0.117422	1.870048	−0.389302
H	2.513941	1.421847	0.297295
Br	0.052968	1.452626	2.066733
**IM3a**, Def2TZVPP	*x*	*y*	*z*
B	−0.645701	−0.266145	0.000516
C	0.298056	0.993464	0.104246
C	1.734946	0.818096	−0.166455
Br	2.188988	0.163002	−1.867974
Br	−0.031400	−2.004684	0.503146
Br	−2.437966	−0.042801	−0.633391
H	−0.117411	1.856241	−0.420917
H	2.515822	1.357364	0.346275
Br	0.036775	1.472353	2.035686
**IM3a**, aug-cc-pVTZ	*x*	*y*	*z*
B	−0.645771	−0.265915	0.000139
C	0.298841	0.992940	0.103064
C	1.735746	0.818788	−0.165383
Br	2.191370	0.158694	−1.866230
Br	−0.033108	−2.006641	0.501459
Br	−2.439672	−0.041665	−0.632155
H	−0.116386	1.856905	−0.419593
H	2.515074	1.364055	0.342608
Br	0.036015	1.469728	2.037221
**TS3′b**, 6-31+G* (B, C, H)/SVP (Br)	*x*	*y*	*z*
B	−0.875823	−0.045623	−0.171688
C	0.073686	1.221086	−0.082103
C	1.514809	1.002473	−0.346574
Br	1.970886	0.508181	−2.103791
Br	−0.252827	−1.775691	0.346630
Br	−2.666220	0.171472	−0.812454
H	−0.329930	2.067703	−0.649731
H	2.328483	1.342642	0.280259
Br	−0.179864	1.768117	1.829142
**TS3′b**, Def2TZVPP	*x*	*y*	*z*
B	−0.874215	−0.040635	−0.172706
C	0.079680	1.214314	−0.088688
C	1.515850	1.009721	−0.346216
Br	1.985775	0.474998	−2.082256
Br	−0.255415	−1.779538	0.322076
Br	−2.672835	0.187583	−0.786023
H	−0.323974	2.061914	−0.646365
H	2.316723	1.389504	0.266360
Br	−0.188390	1.742501	1.823507

**TS3′b**, aug-cc-pVTZ	*x*	*y*	*z*
B	−0.876892	−0.035338	−0.157250
C	0.081428	1.217964	−0.102742
C	1.517078	1.002953	−0.351089
Br	1.989651	0.436078	−2.077247
Br	−0.262332	−1.767188	0.370819
Br	−2.677236	0.185375	−0.771567
H	−0.318327	2.052616	−0.681531
H	2.319453	1.375113	0.263258
Br	−0.189624	1.792787	1.797038

**(*E*)-1 + Br•**, 6-31+G* (B, C, H)/SVP (Br)	*x*	*y*	*z*
B	0.506348	0.929030	0.321079
C	1.998657	0.634471	−0.025554
C	2.945216	0.356716	0.944462
Br	2.064691	3.064376	−0.290783
Br	−0.084079	1.175427	2.138899
Br	−0.817065	0.722232	−1.060308
H	2.246274	0.393833	−1.056404
H	2.776229	0.501177	2.005814
Br	4.662688	−0.234993	0.525005

**(*E*)-1 + Br•**, Def2TZVPP	*x*	*y*	*z*
B	0.094222	0.016473	0.168668
C	0.974154	1.296248	0.193959
C	2.313479	1.285534	−0.111850
Br	0.900616	0.973414	2.621599
Br	0.855986	−1.740248	−0.023247
Br	−1.814258	0.211498	0.047495
H	0.467707	2.252829	0.200726
H	2.895572	0.381106	−0.210811
Br	3.303831	2.847585	−0.327137

**(*E*)-1 + Br•**, aug-cc-pVTZ	*x*	*y*	*z*
B	0.505838	0.915111	0.325383
C	1.995268	0.634504	−0.016035
C	2.940387	0.361147	0.942262
Br	2.092494	3.066007	−0.297764
Br	−0.094381	1.173416	2.137096
Br	−0.814089	0.718600	−1.060125
H	2.240232	0.399547	−1.043516
H	2.776162	0.496842	2.000632
Br	4.657051	−0.222903	0.514278

7. Vibrational frequencies for (*E*)-1 + Br• and ^{add}(*E*)-1 + Br•

Table S10: MP2 and B3LYP-GD3BJ frequencies in cm^{-1} for **(*E*)-1 + Br$^{\bullet}$** with using the 6-31+G*/SVP basis set.

MP2			B3LYP-GD3BJ		
27.8	47.9	65.1	40.0	47.1	64.6
81.9	134.3	152.7	86.6	132.3	167.9
230.1	288.8	437.6	210.9	228.1	290.4
513.3	528.9	731.0	354.9	498.6	713.6
873.5	975.1	1 099.8	762.3	838.9	978.1
1 266.9	1 327.2	1 482.0	1 071.2	1 242.6	1 287.8
3 206.3	3 259.0	6 899.2	1 467.1	3 206.0	3 243.6

MP2

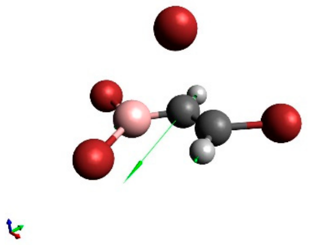

3 206.3 cm^{-1}

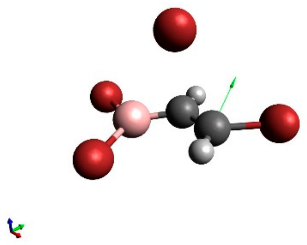

3 259.0 cm^{-1}

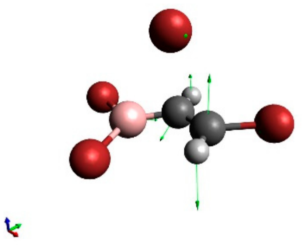

6 899.2 cm^{-1}

B3LYP-GD3BJ

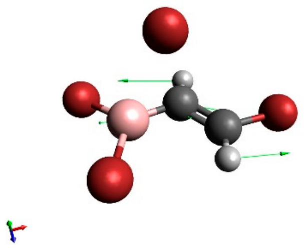

1 467.1 cm^{-1}

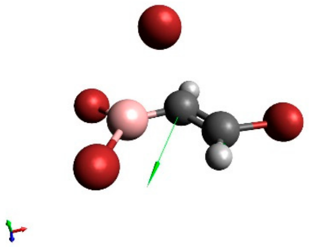

3 206.0 cm^{-1}

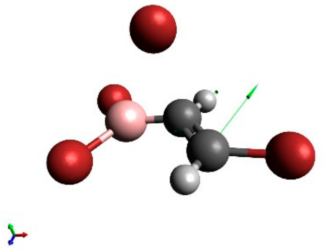

3 243.6 cm^{-1}

Table S11: MP2 and B3LYP-GD3BJ frequencies in cm^{-1} for ^{add}**(*E*)-1 + Br**$^{\bullet}$ with using the 6-31+G*/SVP basis set.

MP2			B3LYP-GD3BJ		
13.2	20.9	46.7	16.3	24.9	78.5
69.1	81.5	123.1	84.1	109.6	140.2
142.6	228.7	294.5	160.8	223.2	292.2
454.6	517.7	720.2	450.1	504.5	686.1
778.4	864.9	969.8	818.6	832.4	983.8
1 136.5	1 277.2	1 346.8	1 098.1	1 253.3	1 330.7
1 640.8	3 216.4	3 265.0	1 605.2	3 188.3	3 251.6

MP2

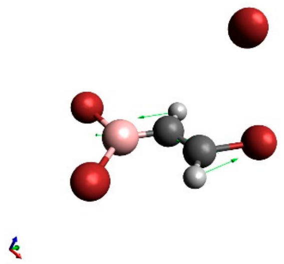

1 640.8 cm^{-1}

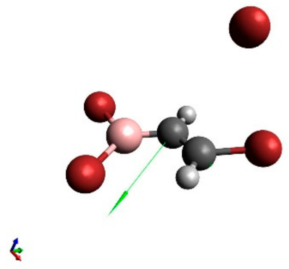

3 216.4 cm^{-1}

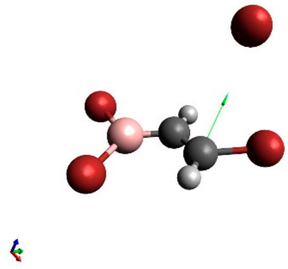

3 265.0 cm^{-1}

B3LYP-GD3BJ

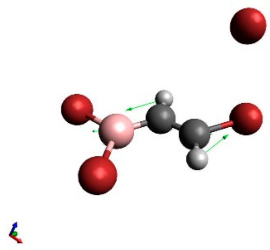

1 605.2 cm^{-1}

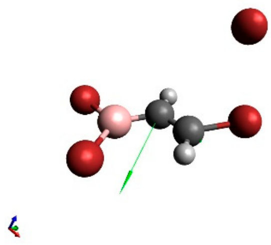

3 188.3 cm^{-1}

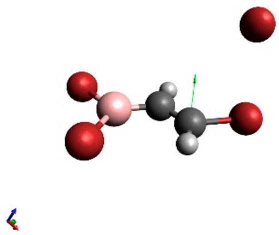

3 251.6 cm^{-1}
